# Safety of Withholding Perioperative Hydrocortisone for Patients With Pituitary Adenomas With an Intact Hypothalamus-Pituitary-Adrenal Axis: A Randomized Clinical Trial

**DOI:** 10.1001/jamanetworkopen.2022.42221

**Published:** 2022-11-16

**Authors:** Xiaopeng Guo, Duoxing Zhang, Haiyu Pang, Zihao Wang, Lu Gao, Yu Wang, Wenbin Ma, Wei Lian, Bing Xing

**Affiliations:** 1 Department of Neurosurgery, Key Laboratory of Endocrinology of Ministry of Health, China Pituitary Adenoma Specialist Council, Peking Union Medical College Hospital, Chinese Academy of Medical Sciences and Peking Union Medical College Hospital, Dongcheng District, Beijing, China; 2 Peking Union Medical College, Tsinghua University, Dongcheng District, Beijing, China; 3 Medical Research Center, State Key Laboratory of Complex Severe and Rare Diseases, Peking Union Medical College Hospital, Chinese Academy of Medical Sciences and Peking Union Medical College Hospital, Dongcheng District, Beijing, China

## Abstract

**Importance:**

Pituitary adenoma is the second most common primary brain tumor. Perioperative hydrocortisone has been used for decades to avoid postoperative adrenal insufficiency. Recent studies suggest that withholding perioperative hydrocortisone may be safe for patients with an intact hypothalamus-pituitary-adrenal (HPA) axis.

**Objective:**

To assess the safety of withholding hydrocortisone during the perioperative period of pituitary adenoma surgery for patients with an intact HPA axis.

**Design, Setting, and Participants:**

A parallel-group, triple-masked, noninferiority randomized clinical trial was conducted at Peking Union Medical College Hospital from November 1, 2020, to January 31, 2022, among 436 patients aged 18 to 70 years with an intact HPA axis undergoing surgery for pituitary adenomas.

**Interventions:**

Hydrocortisone supplementation protocol (intravenous and subsequent oral hydrocortisone, using a taper program) or no-hydrocortisone protocol.

**Main Outcomes and Measures:**

The primary outcome was the incidence of new-onset adrenal insufficiency (morning cortisol level, <5 μg/dL with adrenal insufficiency–related symptoms) during the perioperative period (on the day of operation and the following 2 days). The secondary outcome was the incidence of adrenal insufficiency in postoperative month 3. Analysis was on an intention-to-treat basis.

**Results:**

Of the 436 eligible patients, 218 were randomly assigned to the hydrocortisone group (136 women [62.4%]; mean [SD] age, 45.4 [13.0] years) and 218 to the no-hydrocortisone group (128 women [58.7%]; mean [SD] age, 44.5 [13.8] years). All patients completed 3-month postoperative follow-up. The incidence of new-onset adrenal insufficiency during the perioperative period was 11.0% (24 of 218; 95% CI, 6.9%-15.2%) in the no-hydrocortisone group and 6.4% (14 of 218; 95% CI, 3.2%-9.7%) in the hydrocortisone group, with a difference of 4.6% (95% CI, −0.7% to 9.9%), meeting the prespecified noninferiority margin of 10 percentage points. The incidence of adrenal insufficiency at the 3-month follow-up was 3.7% (8 of 218) in the no-hydrocortisone group and 3.2% (7 of 218) in the hydrocortisone group (difference, 0.5%; 95% CI, −3.0% to 3.9%). Incidences of new-onset diabetes mellitus (1 of 218 [0.5%] vs 9 of 218 [4.1%]), hypernatremia (9 of 218 [4.1%] vs 21 of 218 [9.6%]), hypokalemia (23 of 218 [10.6%] vs 34 of 218 [15.6%]), and hypocalcemia (6 of 218 [2.8%] vs 19 of 218 [8.7%]) were lower in the no-hydrocortisone group than in the hydrocortisone group. Lower preoperative morning cortisol levels were associated with higher risks of the primary event (<9.3 µg/dL; odds ratio, 3.0; 95% CI, 1.5-5.9) and the secondary event (<8.8 µg/dL; odds ratio, 7.8; 95% CI, 2.6-23.4) events.

**Conclusions and Relevance:**

This study found that withholding hydrocortisone was safe and demonstrated noninferiority to the conventional hydrocortisone supplementation regimen regarding the incidence of new-onset adrenal insufficiency among patients with an intact HPA axis undergoing pituitary adenomectomy.

**Trial Registration:**

ClinicalTrials.gov Identifier: NCT04621565

## Key Points

**Question:**

Is withholding hydrocortisone during the perioperative period of pituitary adenoma surgery noninferior to the conventional regimen of hydrocortisone supplementation for patients with an intact hypothalamus-pituitary-adrenal (HPA) axis, who account for more than half of patients with pituitary adenomas?

**Findings:**

In this parallel-group, triple-masked, noninferiority randomized clinical trial that included 436 patients with pituitary adenomas who had an intact HPA axis, withholding perioperative hydrocortisone protocol was noninferior to conventional care with respect to the incidence of postoperative new-onset adrenal insufficiency.

**Meaning:**

The results support the withholding of perioperative hydrocortisone for patients with pituitary adenomas with sufficient baseline HPA axis function.

## Teaser

This randomized clinical trial assesses the safety of withholding perioperative hydrocortisone during the perioperative period of pituitary adenoma surgery for patients with an intact hypothalamus-pituitary-adrenal axis.

## Introduction

Pituitary adenoma is the second most common primary brain tumor (17%) and accounts for 90% of sellar lesions in adults, with an annual incidence of 4.4 per 100 000 population in the United States.^1,2,3^ Apart from hypersecretion symptoms, pituitary adenomas cause hypopituitarism by compressing the pituitary gland and increasing sellar pressure.^4^ Hypopituitarism can also be due to intraoperative injury to the pituitary.^4,5^ Adrenal insufficiency, presenting as dysfunction of the hypothalamic-pituitary-adrenal (HPA) axis, is the most lethal subtype of hypopituitarism, leading to lethargy, fever, vomiting, tachycardia, low blood pressure, circulatory failure, and death.^6^ Since 2 patients who died of acute postoperative adrenal insufficiency were reported in the 1950s, stress-dose glucocorticoid replacement therapy has been recommended as the standard of care during the perioperative period of major surgical procedures.^7,8^

Perioperative glucocorticoid supplementation has long been a routine practice for patients with pituitary adenomas.^9,10,11,12^ There is consensus that patients with insufficient baseline HPA axis function or low serum cortisol levels after surgery for Cushing disease need glucocorticoid supplementation.^13,14^ However, according to the nonrandomized studies, withholding perioperative glucocorticoids might be safe and not lead to a higher risk of postoperative adrenal insufficiency and could avoid glucocorticoid-related adverse events.^10,11,15^ Two recent randomized clinical trials suggested that perioperative glucocorticoids would be safely withheld under intensive monitoring of postoperative serum cortisol levels.^16,17^ However, because of the observational nature of nonrandomized studies and the small sample sizes and improper use of glucocorticoids in randomized clinical trials, a well-designed randomized clinical trial with adequate sample size using the physiologic type of glucocorticoids was warranted to address this long-lasting but unsolved clinical concern.^18^

We conducted this randomized clinical trial to investigate whether the new protocol of withholding perioperative hydrocortisone was clinically safe or noninferior to the conventional hydrocortisone supplementation regimen in terms of the incidence of new-onset acute adrenal insufficiency during the perioperative period for patients with an intact HPA axis undergoing pituitary adenomectomy. We hypothesized that the safety profile of the no-hydrocortisone protocol would be noninferior to conventional care.

## Methods

### Trial Overview

This was a single-center, parallel-group, triple-masked, noninferiority randomized clinical trial conducted from November 1, 2020, through January 31, 2022, at Peking Union Medical College Hospital. We followed the Consolidated Standards of Reporting Trials (CONSORT) reporting guideline for randomized clinical trials.^19^ The trial protocol and statistical plan can be found in Supplement 1. This trial was performed in accordance with the Declaration of Helsinki,^20^ was approved by the Peking Union Medical College Hospital institutional review board, and was monitored by the data safety monitoring board at Peking Union Medical College Hospital. All of the patients provided written informed consent. This study was registered with the ClinicalTrials.gov database (NCT04621565).

### Study Participants

Patients were adults of either sex aged from 18 to 70 years with radiologically suspected pituitary adenomas that needed surgical resection via the transsphenoidal approach and whose HPA axis function was intact, defined by a peak serum cortisol level higher than 18 μg/dL (to convert to nanomoles per liter, multiply by 27.588) after the corticotropin stimulation test. Indication for surgery was evaluated by 2 of us (W.L. and B.X.), both of whom had more than 25 years of experience in pituitary surgery, and determination of surgery was made on agreement with patients. Patients with Cushing disease and patients who had already developed adrenal insufficiency before surgery were excluded from the trial.^6^ The study also excluded patients with pituitary apoplexy or other acute pituitary lesions that needed emergency surgery, patients who needed continuous glucocorticoid replacement therapy owing to other diseases, and patients who were pregnant.

### Randomization, Masking, and Data Collection

Eligible patients were randomly assigned, in a 1:1 ratio, to undergo perioperative administration of hydrocortisone (the hydrocortisone group or standard-of-care group [group 2]) or not to undergo such administration (the no-hydrocortisone group or intervention group [group 1]). A statistician generated the randomization sequence before patient recruitment using SPSS Statistics software, version 23.0 (IBM Corp) with a fixed model. The study staff who collected trial data (D.Z., Z.W., L.G., Y.W., and W.M.) and assessed outcomes (X.G. and H.P.), the principal investigators who provided medical care (W.L. and B.X.), and study participants and their families were all blinded to group assignment to prevent selection bias. The executive nurses and neurosurgical residents of the ZS-2608 Trial Team who allocated drugs or placebos knew the treatment assignment but were not involved in other medical care and data interpretation.

### Interventions and Monitoring

**Figure 1.**
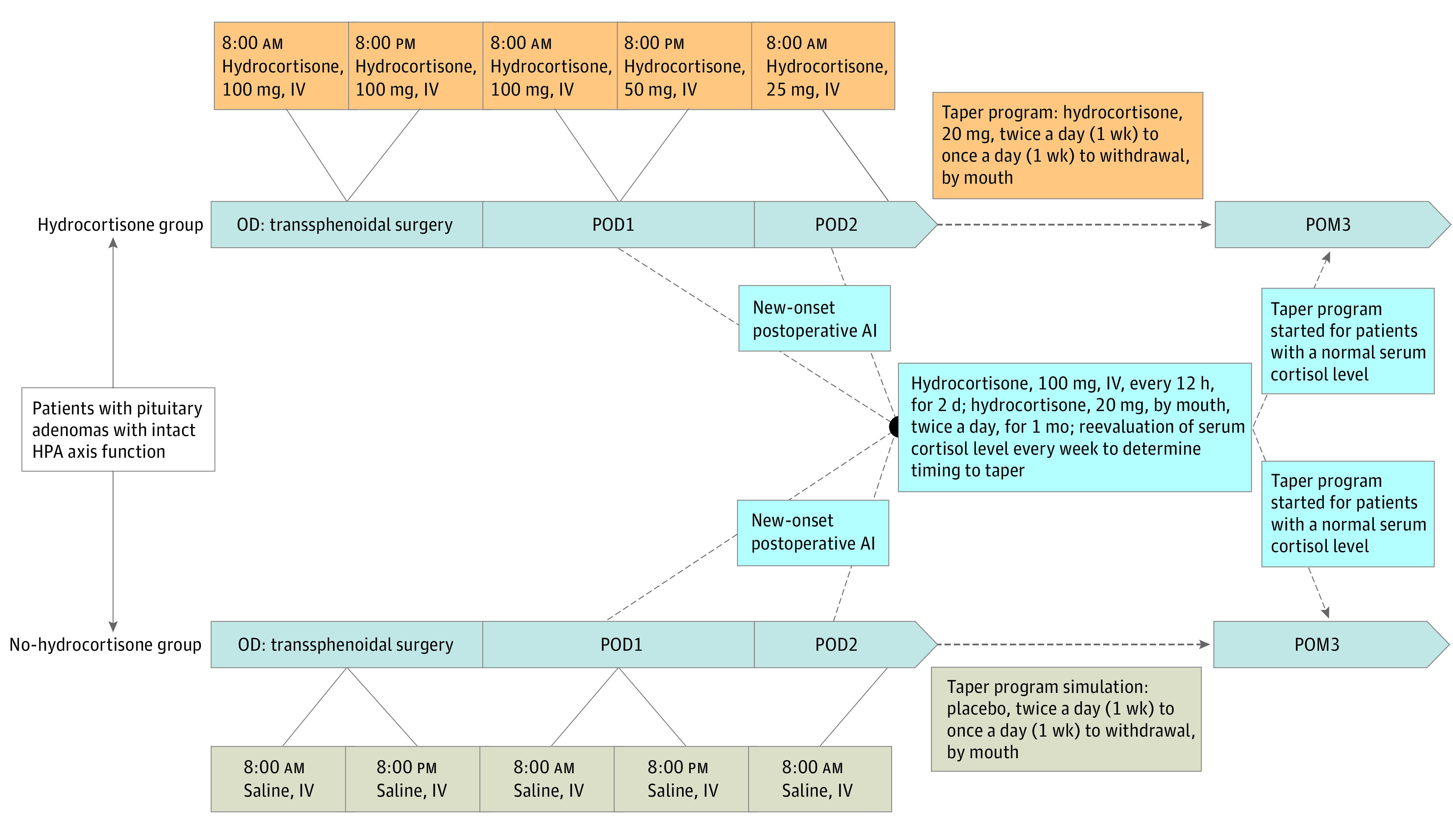
Treatment Protocol for Study Participants In the hydrocortisone (standard-of-care) group, hydrocortisone sodium succinate dissolved in 100 mL of normal saline was given intravenously (IV) on the day of operation (OD; 100 mg at 8 am and 100 mg at 8 pm), postoperative day 1 (POD1; 100 mg at 8 am and 50 mg at 8 pm), and postoperative day 2 (POD2; 25 mg at 8 am). Hydrocortisone tablets were subsequently given orally from the afternoon of POD2 (20 mg, twice a day for 1 week and 20 mg, once a day for another week). In the no-hydrocortisone (intervention) group, patients were given 100 mL of saline, followed by placebo tablets with an identical appearance to the hydrocortisone tablets. On new-onset postoperative adrenal insufficiency (AI) in either group, a modified treatment regimen was used (hydrocortisone sodium succinate, 100 mg, IV, every 12 hours for 2 days, and hydrocortisone tablets, 20 mg, orally, twice a day for 1 month). After these treatments, symptoms and serum cortisol levels were evaluated every week. The taper program was started for patients with a normal morning serum cortisol level. HPA indicates hypothalamic-pituitary-adrenal; POM3, postoperative month 3.

Study interventions are summarized in Figure 1. All patients underwent microscopic pituitary adenomectomy via the transsphenoidal approach. In the standard-of-care group, hydrocortisone sodium succinate dissolved in 100 mL of saline was given intravenously on the day of the operation, postoperative day 1, and postoperative day 2. Supraphysiologic doses of hydrocortisone were used in this period according to the institute experience. Hydrocortisone tablets were subsequently given orally from the afternoon of postoperative day 2 and were stopped at the start of postoperative week 3. This protocol had been widely adopted throughout China and was named the “taper program.” In the no-hydrocortisone group, patients were given 100 mL of saline intravenously, followed by placebo tablets with a similar appearance to the hydrocortisone tablets, simulating the taper program as used in the hydrocortisone group.

Serum cortisol level was evaluated at baseline (8 am), on the day of operation (3 times: after anesthesia induction, after nasal mucosa incision, and after tumor removal), postoperative day 1 (8 am), postoperative day 2 (8 am), and in postoperative month 3 (8 am). The serum adrenocorticotropic hormone (ACTH) level was evaluated before surgery (8 am, baseline), on postoperative day 1 (8 am), and postoperative day 2 (8 am), and in postoperative month 3 (8 am). All blood samples were obtained before hydrocortisone administration.

If new-onset postoperative adrenal insufficiency occurred for patients in either group, the treatment protocol would be rescheduled with a modified hydrocortisone treatment regimen (Figure 1). After these treatments, patient symptoms and serum cortisol levels were evaluated every week. The taper program was started for patients with a normal morning serum cortisol level.

### Primary, Secondary, and Other Prespecified Outcomes

The primary outcome was to assess noninferiority in the no-hydrocortisone group compared with the hydrocortisone group with respect to the incidence of new-onset adrenal insufficiency on the day of the operation, postoperative day 1, and postoperative day 2 (during the perioperative period, as defined in this trial). During the perioperative period, only the first incidence of adrenal insufficiency per patient was counted as the primary end point. The morning cortisol level is mostly used as the first-line test to estimate the development of postoperative adrenal insufficiency because the ACTH stimulation test is not reliable during the early postoperative period.^13,21,22^ Thus, postoperative adrenal insufficiency in this trial was defined as a serum morning cortisol level lower than 5 μg/dL^23^ with at least 1 adrenal insufficiency–related symptom.^6^ The adrenal insufficiency-related symptoms were assessed by 2 of us (Z.W. and L.G.) who were blinded to treatment assignment and did not participate in medical care and data interpretation.

The second outcome was to assess noninferiority of the incidence of adrenal insufficiency in postoperative month 3 in the no-hydrocortisone group compared with the hydrocortisone group. Patients who received a diagnosis of or were receiving treatment for new-onset postoperative adrenal insufficiency in postoperative month 3 were counted for analysis.

Other prespecified outcomes were new-onset diabetes insipidus, bone mineral density loss, deep vein thrombosis, severe infections, diabetes mellitus, and electrolyte disturbance (hyponatremia, hypokalemia, hypocalcemia, hypernatremia, hyperkalemia, and hypercalcemia) during the first 3 months after surgery. These parameters were assessed on postoperative day 1 and postoperative day 2 and at follow-up in postoperative month 3. Urine volume higher than 300 mL/h for more than 3 consecutive hours, urine specific gravity less than 1.005, and serum sodium level higher than 145 mEq/L (to convert to millimoles per liter, multiply by 1.0) indicated diabetes insipidus.^24^ Diabetes mellitus was diagnosed if the patient had a fasting plasma glucose level of 126 mg/dL or higher (to convert to millimoles per liter, multiply by 0.0555) or a 2-hour plasma glucose level of 200 mg/dL or higher after the oral glucose tolerance test.^25^ Bone mineral density and deep vein thrombosis were assessed by dual-energy x-ray absorptiometry and venous ultrasonography.^26,27^ Electrolyte disturbance in this trial was defined as lower or higher than normal levels of serum sodium (135-145 mEq/L), potassium (3.5-5.5 mEq/L [to convert to millimoles per liter, multiply by 1.0]), and calcium (8.52-10.8 mg/L [to convert to millimoles per liter, multiply by 0.25]).

### Statistical Analysis

The sample size was calculated based on the between-group comparison of the primary outcome to detect a 10% difference in the incidence of adrenal insufficiency during the perioperative period. The noninferiority margin (δ) was set as 0.1 based on the results from a prior study.^16^ The calculation formula for qualitative data in noninferiority trials (*N* = 2 × [*U*_α_ + *U*_β_]^2^ × *P*[1 − *P*]/δ^2^) was used, with α set at .05 and β at 0.2. According to the published literature,^16,17^ we set σ as 0.2. Assuming a 10% loss to follow-up, the sample size was finally determined as 218 participants in each trial group.

No participants had been lost to follow-up, and intention-to-treat analysis was used for all end points. Qualitative data were presented as numbers, percentages, and 95% CIs and quantitative data as mean (SD) values. Analyses of the primary, secondary, and other outcomes were performed by calculating the 95% CI of the difference: mean incidence (no-hydrocortisone group) minus mean incidence (hydrocortisone group). Noninferiority was identified if the upper limit of the 95% CI was smaller than the margin of 10 percent points. All *P* values were from 2-sided tests, and results were deemed statistically significant at *P* < .05. We assessed superiority for the outcomes in which the noninferiority was achieved. Post hoc subgroup analyses and prognostic analyses were performed for the primary and secondary outcomes. We used the *t* test for comparisons of continuous variables and the χ^2^ test for categorical variables. We used the receiver operating characteristic curve to detect the cutoff value of baseline serum cortisol in determining early and late postoperative adrenal insufficiency. All analyses were performed using SPSS Statistics, version 23.0 (IBM Corp). Graphs were generated using Prism, version 8.4.3 (GraphPad Software).

## Results

### Trial Population

**Figure 2.**
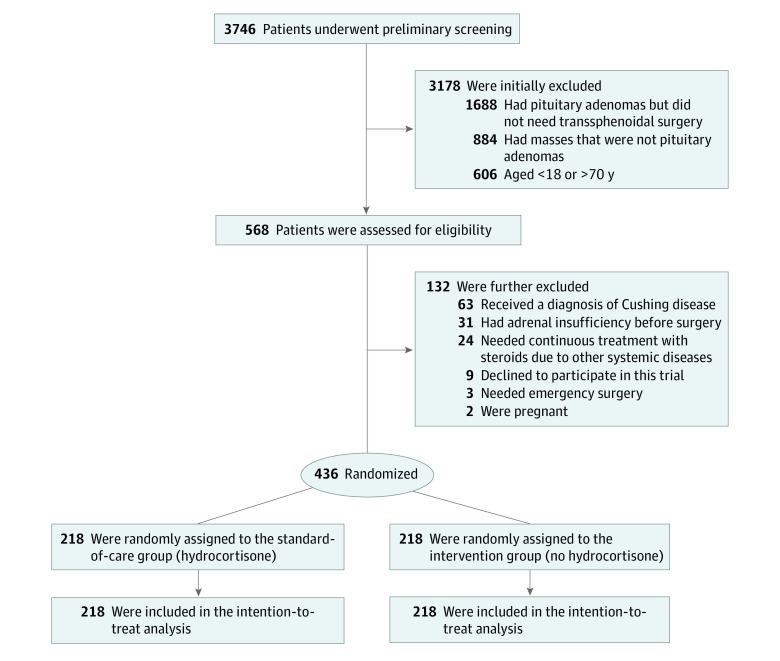
Study Flow Diagram

A total of 436 eligible patients (264 women [60.6%] and 172 [39.4%] men; mean [SD] age, 45 [13] years) were enrolled and underwent randomization (Figure 2). Of the 436 eligible patients, 218 were randomly assigned to the hydrocortisone group (136 women [62.4%]; mean [SD] age, 45.4 [13.0] years) and 218 to the no-hydrocortisone group (128 women [58.7%]; mean [SD] age, 44.5 [13.8] years) (Table). The clinical characteristics were well balanced between the 2 trial groups except for the incidence of hypopituitarism in other than HPA axes (128 of 218 [58.7%] in the hydrocortisone group and 106 of 218 [48.6%] in the no-hydrocortisone group). All samples were pathologically confirmed as pituitary adenomas, and all pateints completed 3-month postoperative follow-up.

**Table.** Characteristics of the Patients

Characteristic	Patients, No. (%)	
	No hydrocortisone (n = 218)	Hydrocortisone (n = 218)
Age, y		
Mean (SD)	44.5 (13.8)	45.4 (13.0)
<45	109 (50.0)	105 (48.2)
≥45	109 (50.0)	113 (51.8)
Sex		
Male	90 (41.3)	82 (37.6)
Female	128 (58.7)	136 (62.4)
Height, mean (SD), cm	166.8 (8.8)	166.5 (7.9)
Weight, mean (SD), kg	71.3 (13.6)	70.8 (13.3)
BMI, mean (SD)	25.5 (3.8)	25.4 (3.5)
Preoperative comorbidities		
Hypertension	56 (25.7)	59 (27.1)
Diabetes mellitus	18 (8.3)	26 (11.9)
Deep vein thrombosis	0	2 (0.9)
Bone mineral density loss	31 (14.2)	32 (14.7)
Others	129 (59.2)	140 (64.2)
Radiologic features of tumors		
Maximal diameter, mean (SD), mm	20.5 (8.6)	20.6 (7.9)
Macroadenoma	196 (89.9)	200 (91.7)
Microadenoma	22 (10.1)	18 (8.3)
Cavernous sinus invasion	82 (37.6)	76 (34.9)
Tumor type		
Nonfunctioning	134 (61.5)	127 (58.3)
Functioning	84 (38.5)	91 (41.7)
Growth hormone	51 (23.4)	50 (22.9)
Prolactin	30 (13.8)	32 (14.7)
Thyroid stimulating hormone	3 (1.4)	4 (1.8)
Plurihormonal	0	5 (2.3)
Baseline HPA axis function		
ACTH, mean (SD), pg/mL	31.2 (21.5)	28.7 (18.1)
Cortisol, mean (SD), μg/dL	13.3 (5.3)	13.2 (6.1)
Preoperative hypopituitarism	106 (48.6)	128 (58.7)
Degree of tumor resection		
Gross total	136 (62.4)	146 (67.0)
Subtotal	82 (37.6)	72 (33.0)

Abbreviations: ACTH, adrenocorticotropic hormone; BMI, body mass index (calculated as weight in kilograms divided by height in meters squared); HPA, hypothalamic-pituitary-adrenal.

SI conversion factors: to convert ACTH to picomoles per liter, multiply by 0.22; to convert cortisol to nanomoles per liter, multiply by 27.588.

### Primary End Point

**Figure 3.**
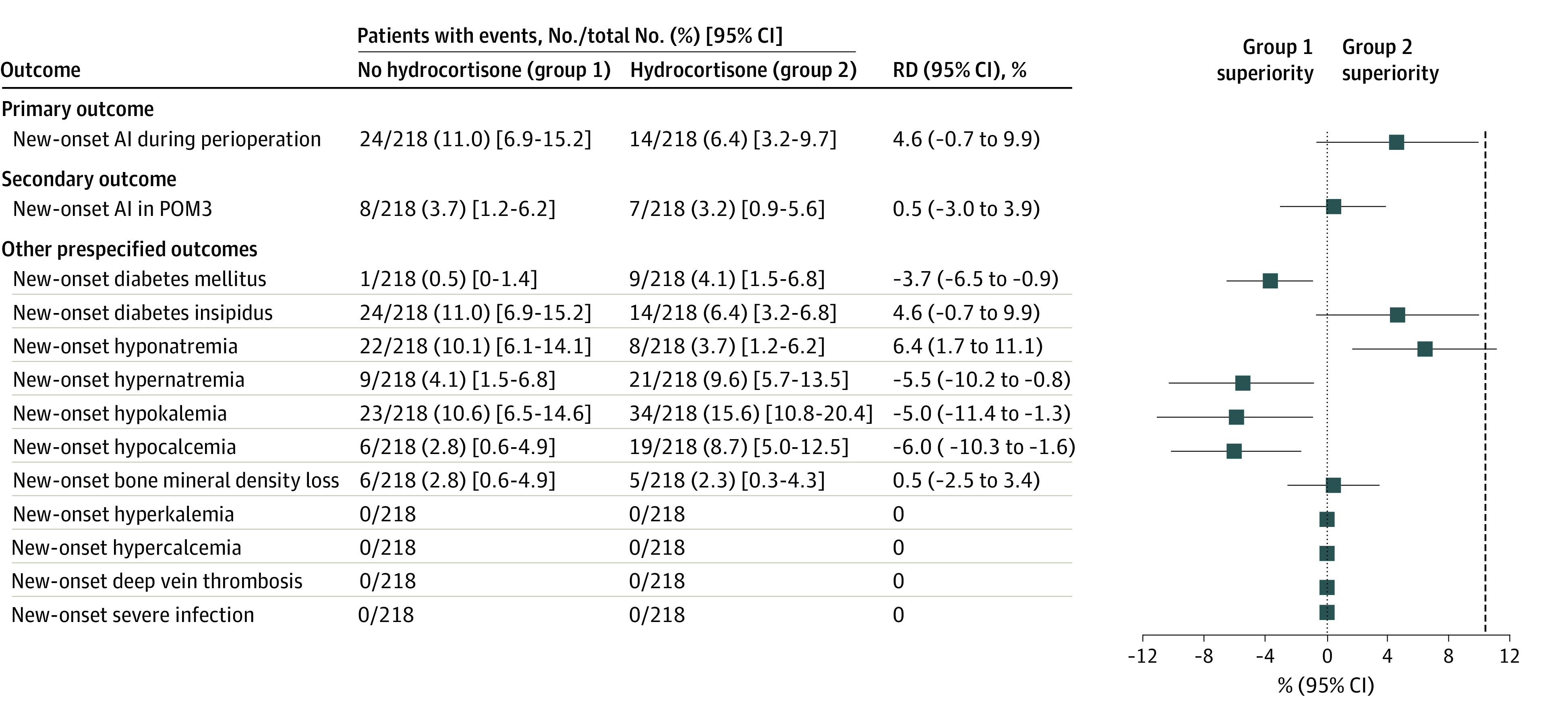
Primary, Secondary, and Other Prespecified Outcomes According to Trial Group Analyses for all end points were based on the intention-to-treat population. The primary outcome was new-onset postoperative adrenal insufficiency (AI) within postoperative day 2. The prespecified noninferiority margin was 10 percentage points (group 1 noninferiority [dotted line]). The secondary outcome was active postoperative AI in postoperative month 3 (POM3). Additional outcomes included surgical- and glucocorticoid-related adverse events during the first 3 months after surgery. Incidence (95% CI) differences were carried to 1 decimal place. RD indicates risk difference.

The incidence of new-onset adrenal insufficiency during the perioperative period was 11.0% (24 of 218; 95% CI, 6.9%-15.2%) in the no-hydrocortisone group and 6.4% (14 of 218; 95% CI, 3.2%-9.7%) in the hydrocortisone group (difference, 4.6%; 95% CI, −0.7% to 9.9%). Because the upper limit of the 95% CI was smaller than the predetermined noninferiority margin of 10 percentage points, noninferiority was achieved for the nonuse of hydrocortisone (Figure 3). Of the 38 patients who had a primary event (eTable 1 in Supplement 2), 35 (92.1%) normalized their HPA axis function at the 3-month follow-up.

### Secondary End Point

The incidence of adrenal insufficiency in postoperative month 3 was 3.7% (8 of 218) in the no-hydrocortisone group and 3.2% (7 of 218) in the hydrocortisone group (difference, 0.5%; 95% CI, −3.0% to 3.9%). Thus, noninferiority was achieved for the secondary outcome (Figure 3). Of the 15 patients with adrenal insufficiency in postoperative month 3 (eTable 2 in Supplement 2), 3 patients developed new-onset adrenal insufficiency within the perioperative period and the other 12 patients developed new-onset adrenal insufficiency after the perioperative period.

### Post Hoc Subgroup Analyses

Although the incidence of the primary event when using the no-hydrocortisone protocol tended to be higher than that when using the hydrocortisone regimen in most subgroups, the differences were not statistically significant (eTable 3 in Supplement 2). Among patients with a body mass index less than 25.5 (calculated as weight in kilograms divided by height in meters squared), the incidence of adrenal insufficiency in the no-hydrocortisone group (14 of 114 [12.3%]) was significantly higher than in the hydrocortisone group (3 of 121 [2.5%]; *P* = .004). Post hoc analyses of most subgroups were consistent with the main findings of the secondary end point except for the subgroup of patients with microadenoma (1 of 18 [5.6%; 95% CI, 0%-16.1%] in the hydrocortisone group and 1 of 22 [4.5%; 95% CI, 0%-13.2%] in the no-hydrocortisone group) (eTable 4 in Supplement 2).

### Dynamic Changes in Serum Cortisol and ACTH Levels

**Figure 4.**
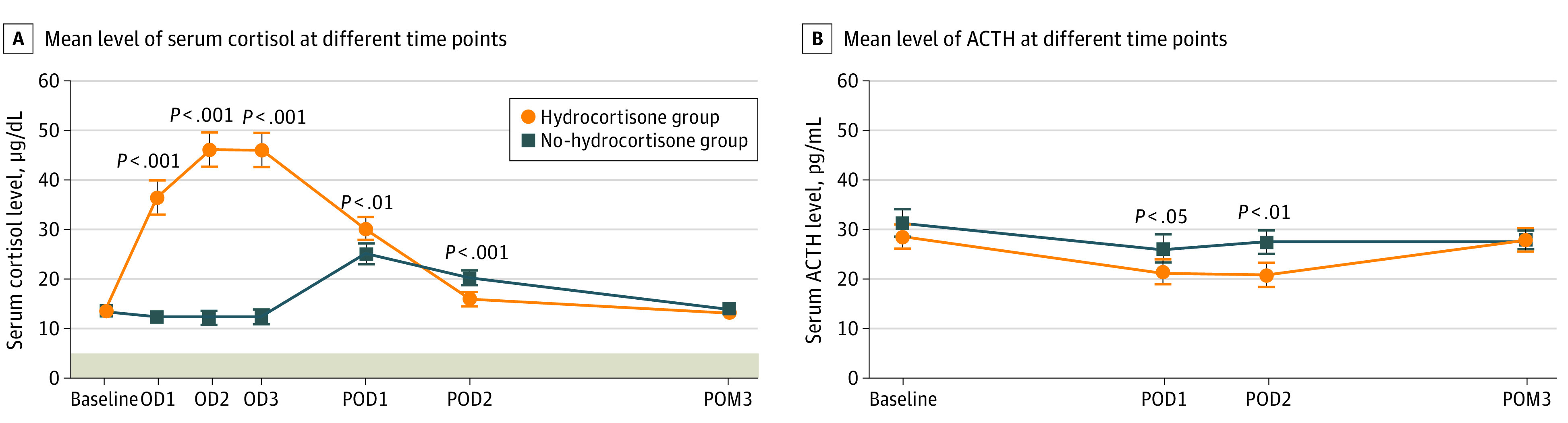
Levels of Serum Cortisol and Adrenocorticotropic Hormone (ACTH) Over Time After Transsphenoidal Surgery The connecting lines illustrate the patterns of dynamic changes in hypothalamic-pituitary-adrenal axis hormones. OD1 indicates day of operation after anesthesia induction; OD2, day of operation after nasal mucosa incision; OD3, day of operation after tumor removal; POD1, postoperative day 1; POD2, postoperative day 2; and POM3, postoperative month 3. Error bars indicate 95% CIs. SI conversion factors: to convert ACTH to picomoles per liter, multiply by 0.22; to convert cortisol to nanomoles per liter, multiply by 27.588.

In the no-hydrocortisone group, compared with the mean (SD) baseline serum cortisol level (13.3 [5.3] μg/dL), the mean (SD) serum cortisol level decreased on the day of the operation (after anesthesia induction, 12.3 [5.6] μg/dL; *P* = .01; after nasal mucosa incision, 12.2 [9.8] μg/dL; *P* = .10; and after tumor removal, 12.5 [9.7] μg/dL; *P* = .18), increased on postoperative day 1 (25.1 [15.1] μg/dL; *P* < .001) and postoperative day 2 (20.2 [10.0] μg/dL; *P* < .001), and returned to the baseline level at postoperative month 3 (13.9 [5.4] μg/dL; *P* = .30) (Figure 4).

In the hydrocortisone group, compared with the mean (SD) baseline serum cortisol level (13.2 [6.1] µg/dL), mean (SD) serum cortisol levels increased on the day of the operation (after anesthesia induction, 36.3 [25.7] μg/dL; *P* < .001; after nasal mucosa incision, 46.0 [25.0] μg/dL; *P* < .001; and after tumor removal, 46.1 [24.8] μg/dL; *P* < .001) and postoperative day 1 (30.1 [16.6] μg/dL; *P* < .001), decreased but were still higher than baseline on postoperative day 2 (16.0 [8.9] μg/dL; *P* < .001), and decreased further in postoperative month 3 (13.2 [5.4] μg/dL; *P* = .49). Reduction of mean (SD) ACTH levels was statistically significant in the hydrocortisone group compared with the no-hydrocortisone group (baseline, 28.7 [18.1] vs 31.2 [21.5] µg/dL; postoperative day 1, 21.4 [18.1] vs 26.2 [21.4] µg/dL; postoperative day 2, 20.8 [17.6] vs 27.5 [18.6] µg/dL; and postoperative month 3, 28.0 [16.7] vs 27.7 [15.6] µg/dL) (Figure 4).

### Other Prespecified Outcomes

Nine patients (4.1%) in the hydrocortisone group and 1 patient (0.5%) in the no-hydrocortisone group developed diabetes mellitus after surgery (difference, –3.7%; 95% CI, −6.5% to −0.9%), achieving noninferiority as well as superiority in the no-hydrocortisone group (Figure 3). Regarding the incidences of acute postoperative hypernatremia (21 of 218 [9.6%] in the hydrocortisone group vs 9 of 218 [4.1%] in the no-hydrocortisone group; difference, –5.5%; 95% CI, −10.2% to −0.8%), hypokalemia (34 of 218 [15.6%] in the hydrocortisone group vs 23 of 218 [10.6%] in the no-hydrocortisone group; difference, –5.0%; 95% CI, −11.4% to −1.3%), and hypocalcemia (19 of 218 [8.7%] in the hydrocortisone group vs 6 of 218 [2.8%] in the no-hydrocortisone group; difference, –6.0%; 95% CI, −10.3% to −1.6%), the no-hydrocortisone protocol also achieved superiority. These electrolyte disturbances were not detected in all patients at the 3-month follow-up. New-onset postoperative diabetes insipidus (14 of 218 [6.4%] in the hydrocortisone group vs 24 of 218 [11.0%] in the no-hydrocortisone group; difference, 4.6%; 95% CI, −0.7% to 9.9%) and bone mineral density loss (5 of 218 [2.3%] in the hydrocortisone group vs 6 of 218 [2.8%] in the no-hydrocortisone group; difference, 0.5%; 95% CI, −2.5% to 3.4%) achieved noninferiority in the no-hydrocortisone group. Diabetes insipidus was transient in most patients, while continuous disease was found in 2 patients (0.9%) in the hydrocortisone group and 3 patients (1.4%) in the no-hydrocortisone group at the 3-month follow-up. Transient hyponatremia was found in 8 patients (3.7%) in the hydrocortisone group and 22 patients (10.1%) in the no-hydrocortisone group (difference, 6.4%; 95% CI, 1.7%-11.1%), and all sodium levels were restored at the 3-month follow-up.

### Prognostic Factors for Event Occurrence

A higher risk of the primary event was observed for patients with a baseline cortisol level lower than 9.3 μg/dL (odds ratio [OR], 3.0; 95% CI, 1.5-5.9; *P* = .001; eTable 5 and eFigure 1 in Supplement 2). A higher risk of the secondary event was observed for patients with a baseline cortisol level lower than 8.8 μg/dL (OR, 7.8; 95% CI, 2.6-23.4; *P* < .001; eTable 6 and eFigure 2 in Supplement 2). Whether patients had preoperative hormone deficiency in other axes than the HPA axis did not have an effect on event occurrence (eTable 5 and eTable 6 in Supplement 2). The adjusted incidence difference for the primary outcome using Cochran-Mantel-Haenszel weighting for preoperative cortisol level and body mass index was 4.5% (95% CI, −0.8% to 9.8%), also achieving noninferiority (eTable 7 in Supplement 2).

## Discussion

The present randomized clinical trial evaluated the safety profile of withholding perioperative hydrocortisone for patients with pituitary adenomas who had an intact HPA axis, with an ultimate goal of minimizing glucocorticoid overuse. The trial demonstrated noninferiority of the no-hydrocortisone protocol regarding new-onset adrenal insufficiency during the perioperative period compared with the standard hydrocortisone replacement regimen that had been advocated for decades worldwide.^10,28,29,30,31^ To our knowledge, our study has the largest sample size of its kind and, for the first time, sets postoperative adrenal insufficiency as the primary end point to address the long-lasting but still unsolved clinical problem by using a well-designed, parallel-group, triple-masked, noninferiority randomized clinical trial.

Although nonrandomized studies have proposed that withholding perioperative glucocorticoid administration may be safe, it was not until 2019 and 2020 that 2 small randomized clinical trials drew supporting conclusions.^16,17^ However, postoperative adrenal insufficiency was not set as the primary end point in either trial, leading to a restricted ability to evaluate the safety of the new treatment protocol.

Dexamethasone can be used during the induction of anesthesia to decrease the risk of postoperative nausea and vomiting,^32^ but is not a necessity. In our trial, dexamethasone was prohibited to avoid suppression of the HPA axis and subsequent glucocorticoid replacement. Moreover, the taper program was adopted to avoid glucocorticoid withdrawal syndrome and unexpected adrenal insufficiency.^14^ The cortisol level rebounded almost 2-fold on postoperative days 1 and 2, showing stress tolerance for pituitary surgery and a subsequent robust intrinsic response in these patients.^10,11,33,34^

For patients with pituitary adenomas and an intact HPA axis, the literature reports that early postoperative adrenal insufficiency occurs in fewer than 25% of patients and is sustained in fewer than 20% of patients.^11,16,17,35,36,37^ We observed that fewer than 10% of patients developed early postoperative adrenal insufficiency, and most normalized their HPA axis function during the first 3 postoperative months after short-term hydrocortisone therapy. Although patients in the no-hydrocortisone group showed a higher tendency of developing adrenal insufficiency compared with those using hydrocortisone, noninferiority was achieved for the primary end points, and the incidences of the second end point were not significantly different between the 2 groups.

Neither group had a glucocorticoid- or surgery-related complication with an incidence of greater than 20% during the first 3 months after surgery, and most of the complications were reversible, confirming the safety profile of withholding perioperative hydrocortisone in terms of adverse events. Although glucocorticoid supplementation might unmask diabetes insipidus,^38,39^ its incidences were not different between the 2 groups, concordant with other studies.^11,17^ Hydrocortisone can influence the levels of blood glucose and several electrolytes.^40^ Our result confirmed that the no-hydrocortisone protocol was better to decrease the risks of postoperative hypernatremia, hypokalemia, hypocalcemia, and diabetes mellitus. However, patients should be intensively monitored for postoperative hyponatremia when the new no-hydrocortisone protocol is used.

Among patients with a body mass index less than 25.5, early postoperative adrenal insufficiency occurred in 2.5% of patients using hydrocortisone, significantly lower than the 12.3% of patients in the no-hydrocortisone group. One explanation was that lean patients with a low metabolism of cortisol who received hydrocortisone supplementation had a relatively high cortisol level beyond the actual endogenous production ability.

### Limitations

This study has some limitations. Data regarding the long-term safety of the nonuse of hydrocortisone are scarce, and our results with a 3-month follow-up should be confirmed with further studies with a longer follow-up period. Moreover, the high dose of hydrocortisone used during the perioperative period was based on our institutional practice, the protocol of which might be variable among different neurosurgical centers. Greater adoption of the no-hydrocortisone protocol will assist in the generalizability of results in the clinical setting and accumulation of more safety data.

## Conclusions

This randomized clinical trial involving patients with pituitary adenomas who had an intact HPA axis demonstrated that nonuse of perioperative hydrocortisone is clinically safe after intense hormone monitoring regarding the incidence of postoperative new-onset adrenal insufficiency. Thus, the result of this trial supports the nonuse of hydrocortisone during the perioperative period of pituitary adenomectomy for patients with sufficient HPA axis function.

## Notes

Supplement 1.
Trial Protocol

Supplement 2. **eTable 1.** List of the Patients With a Primary Event **eTable 2.** List of the Patients With a Secondary Event **eTable 3.** Subgroup Analyses of the Primary Outcome in the Intention-to-Treat Population **eTable 4.** Subgroup Analyses of the Secondary Outcome in the Intention-to-Treat Population **eTable 5.** Prognostic Factors Associated With a Primary Event in 436 Patients **eTable 6.** Prognostic Factors Associated With a Secondary Event in 436 Patients **eTable 7.** Adjusted Incidence Rate Difference and 95% CI for the Primary Outcome **eFigure 1.** ROC Curve for Preoperative Cortisol in Predicting AI Within 2 Days After Surgery **eFigure 2.** ROC Curve for Preoperative Cortisol in Predicting AI from POD3 to POM3

Supplement 3.
Nonauthor Collaborators

Supplement 4.
Data Sharing Statement
