# Supplementary material for: Safety of Withholding Perioperative Hydrocortisone for Patients With Pituitary Adenomas With an Intact Hypothalamus-Pituitary-Adrenal Axis: A Randomized Clinical Trial
Source: JAMA Netw Open. 2022 Nov 16;5(11):e2242221. doi: 10.1001/jamanetworkopen.2022.42221 (PMC9669812; doi:10.1001/jamanetworkopen.2022.42221)
Supplement: Supplement 1. — Trial Protocol [file jamanetwopen-e2242221-s001.pdf]

Supplement 1. Trial Protocol

Table of Contents

Original Protocol and Statistical Analysis Plan *2*
1. Brief Study Description 2
2. Study Design 2
3. Background Description 2
4. Arms and Interventions 3
5. Outcome Measures 4
6. Inclusion and Exclusion Criteria 5
7. Sample Size Estimation 6
8. Randomization, Masking, and Data Collection 7
9. Statistical Analysis Plan 7
10. Randomization sequence 9
11. Informed Consent in English and Chinese 14
Final Protocol and Statistical Analysis Plan *22*
12. Brief Study Description 22
13. Study Design 22
14. Background Description 22
15. Arms and Interventions 24
16. Outcome Measures 25
17. Inclusion and Exclusion Criteria 26
18. Sample Size Estimation 28
19. Randomization, Masking, and Data Collection 28
20. Statistical Analysis Plan 29
21. Randomization sequence 30
22. Informed Consent in English and Chinese 35
Summary of Changes to the Protocol and the Statistical Analysis Plan *43*

Original Protocol and Statistical Analysis Plan

1. Brief Study Description

The investigators from Beijing hypothesize that withholding hydrocortisone replacement therapy during the perioperative period in patients with pituitary adenomas whose hypothalamus pituitary adrenal (HPA) axis function are intact are safe as compared with the conventional hydrocortisone replacement therapy.

2. Study Design

Study Type: Interventional (Clinical Trial)

Estimated Enrollment: 436 participants

Allocation: Randomized

Intervention Model: Parallel Assignment

Intervention Model Description: Participants are assigned to one of two groups, whether using hydrocortisone or not during peri-operation, in parallel for the duration of the study.

Masking: Triple (Participant, Investigator, Outcomes Assessor)

Masking Description: Participants, investigators, and outcome assessors are all prevented from having knowledge of the interventions assigned to individual participants. Care provider (nurse and resident) knows the individualized interventions.

3. Background Description

Pituitary is the headquarters of the endocrine system of the body, secreting several hormones, including adrenocorticotropic hormone (ACTH), growth hormone, thyroid stimulating hormone, follicle stimulating hormone, and luteinizing hormone, maintaining the normal function of the endocrine organs throughout the body. After the resection of pituitary adenomas (PAs), pituitary dysfunction (hypopituitarism) is seen in a small proportion of patients. One of the most severe and even life-threatening type is adrenal insufficiency (AI) or pituitary crisis.

Since two cases were reported in the 1950s, 7 decades ago, who died of acute postoperative AI because of withdrawal of continuous glucocorticoids administration, stress-dose glucocorticoid replacement therapy has been recommended as the standard of care during peri-operation of major surgeries, especially for the patients receiving continuous steroids replacement therapy.

Patients undergoing pituitary surgery have been usually given "stress dose" steroids regardless of the function status of their HPA axis. However, results of several retrospective non-randomized studies showed that there was no significantly increase in the incidence of postoperative AI in the no hydrocortisone supplementation group than in the supplementation group. Given the considerable side effects of using steroids, whether hydrocortisone administration is necessary for patients with PAs, especially those with an intact HPA axis, during peri-operation needs to be discussed.

Two recent randomized trials suggested that perioperative steroids would be safely withheld in patients with an intact HPA axis. However, because of the small sample sizes (43 and 40 cases) that were not sufficient to calculate the incidence of postoperative AI and the improper types of glucocorticoids used (dexamethasone and prednisone) in the two trials, a well-designed randomized trial with adequate sample size using the physiological type of steroids (hydrocortisone) is warranted to address this long-lasting but unsolved clinical concern.

Peking Union Medical College Hospital (PUMCH) is the TOP 1 hospital in China for a consecutive of 11 years and is the China Pituitary Disease Registry Center and the lead of the China Pituitary Adenoma Specialist Council. The number of patients with PAs are large and patient compliance is extremely high, setting foundation for the current trial.

Here, the investigators from PUMCH aim to launch a single-center, parallel-group, non-inferiority, randomized, controlled trial to verify the hypothesis that withholding hydrocortisone during the peri-operation in patients with PAs whose HPA axis are intact are safe regarding new-onset postoperative AI as compared with the traditional hydrocortisone supplementation therapy.

4. Arms and Interventions

Arm 1:

Experimental: No-hydrocortisone group (Patients receive no perioperative hydrocortisone)

Intervention/treatment: No hydrocortisone or other steroids are given before, during, and after the surgery, except that the patient develops postoperative AI. If a patient develops postoperative AI, he/she needs to receive hydrocortisone treatment (20mg at 0800 and 20mg at 1600 for one month) and then routinely check the level of morning serum cortisol to decide the optimal timing to start the hydrocortisone taper program.

Arm 2:

Active Comparator: Hydrocortisone group (Patients receive perioperative hydrocortisone)

Intervention/treatment: Hydrocortisone sodium succinate is given on the day of operation (100mg at 0800 & 100mg at 2000), the postoperative day 1 (100mg at 0800 & 50mg at 2000), and the postoperative day 2 (25mg at 0800). Hydrocortisone tablet (po.) is then given starting from the afternoon of the postoperative day 2 (20mg at 1600 and 0800, twice a day) to the end of the first postoperative week, and 20mg at 0800 during the second postoperative week. This is called the "taper program". If a patient develops postoperative AI, he/she needs to stop his/her original protocol and receive hydrocortisone treatment (20mg at 0800 and 20mg at 1600 for one month) and then routinely check the level of morning serum cortisol to decide the optimal timing to start the hydrocortisone taper program.

5. Outcome Measures

1) Primary outcome

- Incidence of newly-onset adrenal insufficiency [During the first 3 postoperative days]

Adrenal insufficiency: Lower-than-normal serum cortisol level at 8 a.m., plus the following related symptoms, including serious fatigue, muscle weakness, decreased appetite, nausea, vomiting, diarrhea, low blood pressure, palpitation, and fever.

2) Secondary outcome

- Incidence of newly-onset adrenal insufficiency [From the 3rd postoperative day to the 3rd postoperative month]

Adrenal insufficiency: Lower-than-normal serum cortisol level at 8 a.m., plus the following related symptoms, including serious fatigue, muscle weakness, decreased appetite, nausea, vomiting, diarrhea, low blood pressure, palpitation, and fever.

3) Other Outcome Measures:

- Rate of newly-developed diabetes mellitus [Time Frame: During the first 3 postoperative months]

  Diabetes mellitus: A random reading of blood sugar level more than 200 mg/dL (11.1 mmol/L), or a reading after two hours (after OGTT) over 200 mg/dL (11.1 mmol/L).

- Rate of newly-developed diabetes insipidus [Time Frame: During the first 3 postoperative months]

  Diabetes insipidus: Urine volume > 300ml for more than 3h or > 6 liter per day, specific gravity of urine <1.003, and serum sodium levels > 145 mmol/L.

- Concentration of blood electrolytes [Time Frame: At the 3rd postoperative month]

The level of Na+, K+, and Ca++ in the blood.

- Concentration of blood cells [Time Frame: At the 3rd postoperative month]

Numbers of erythrocytes, leukocytes, neutrophils, lymphocytes, thrombocytes in the blood.

- Percentage of blood cells [Time Frame: At the 3rd postoperative month]

Percentage of neutrophils, monocytes, lymphocytes of the leukocytes.

- Concentration of indexes of coagulation function [Time Frame: At the 3rd postoperative month]

Prothrombin time, activated partial thromboplastin time, and thrombin time.

- Level of international normalized ratio [Time Frame: At the 3rd postoperative month]

Level of international normalized ratio.

- Level of D-Dimer [Time Frame: At the 3rd postoperative month]

Level of D-Dimer.

- Rate of deep venous thrombosis [Time Frame: During the first 3 postoperative months]

Deep venous thrombosis is detected by ultrasound.

- Rate of other complications [Time Frame: During the first 3 postoperative months]

Decreased bone density, osteoporosis, fracture, acne, and infections.

6. Inclusion and Exclusion Criteria

Inclusion Criteria:

- Patients with PAs that need transsphenoidal surgery, whose HPA axis are intact
- Patients of either gender aged from 18 years to 70 years

Exclusion Criteria:

- Patients with Cushing's disease

- Patients with PAs who have AI before surgery
- Patients with pituitary apoplexy or other acute pituitary conditions that need emergency surgery
- The postoperative pathology result indicates that the tumor is not a PA
- Patients that refuse to participate or those who ask to quit after enrollment

Patients Flow

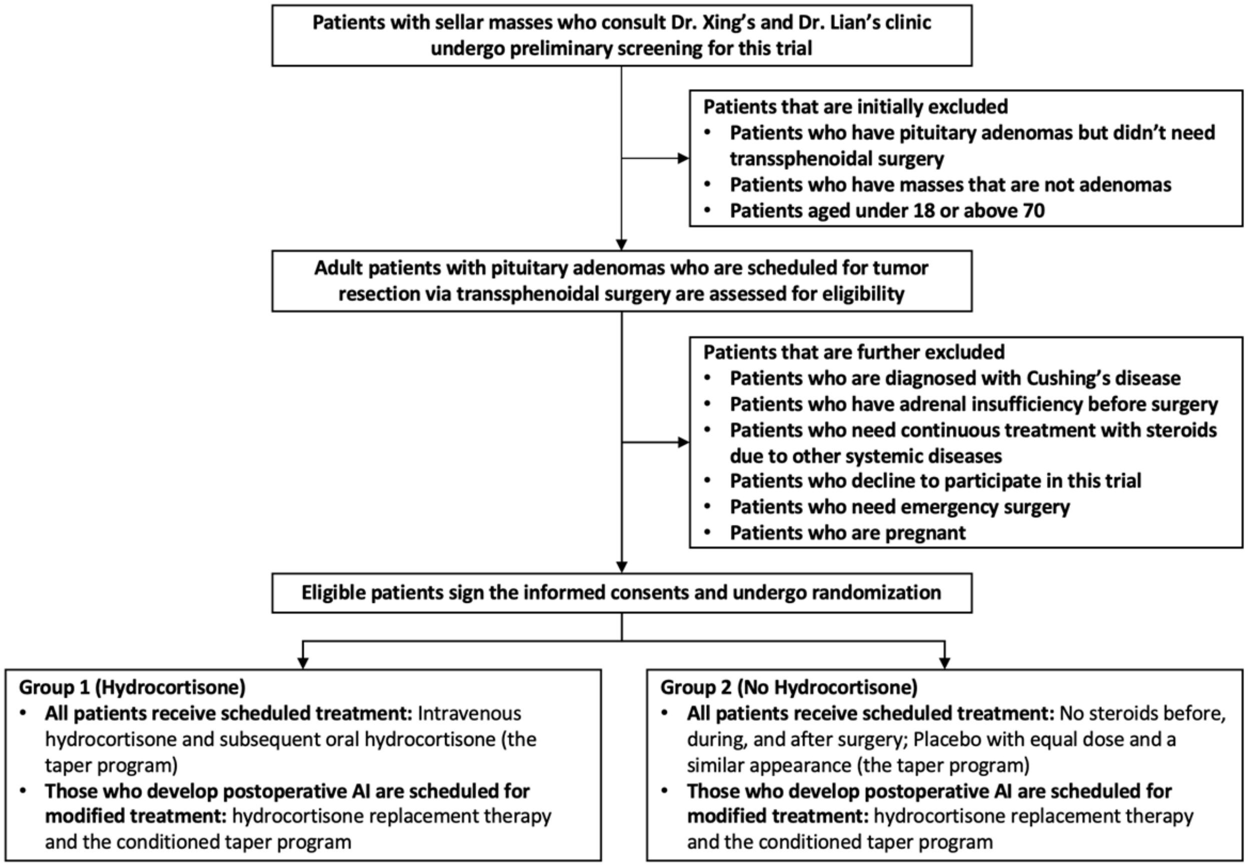

7. Sample Size Estimation

- We design the study as a non-inferiority trial to address the clinical question of whether the no-hydrocortisone protocol is non-inferior to the traditional regimen regarding the incidences of new-onset AI during perioperation after surgery for PAs in patients with an intact HPA-axis.
- The sample size is calculated based on the between-group comparison of the primary outcome (the incidences of new-onset AI during perioperation) to detect a 10% difference on the incidence of AI (as

indicated by the 95% CI) during perioperation of pituitary surgery. The non-inferiority margin (δ) is thus set as 0.1.

- The calculation formula for qualitative data in non-inferiority trials ($N=2\times[U\alpha+U\beta]^2\times P[1-P]/\delta^2$) is used, with α set at 0.05 and β at 0.2.
- According to the published literature (DOI: 10.3171/2019.11.JNS192381; DOI: 10.1093/neuros/nyy479) and our preliminary observation, we set σ as 0.2.
- Assuming a 10% loss to follow-up, the sample size is finally determined as 218 in each trial group and 436 in total.

8. Randomization, Masking, and Data Collection

- Patients are to be randomly assigned, in a 1:1 ratio, to undergo perioperative administration of hydrocortisone or not to undergo such administration.
- The randomization sequence will be generated before patient recruitment using SPSS Statistics software with a fixed model.
- The blinded assignment will be performed.
- The study staff who collected trial data and those who assess outcomes, the investigators, and study participants and their families are all blinded to group assignment before unblinding.
- The executive nurses and neurosurgical residents who allocated drugs or placeboes know the treatment assignment and keep secret.

9. Statistical Analysis Plan

- This is a single-center, parallel-group, non-inferiority, randomized, controlled trial.
- We follow the CONSORT guideline for reporting the result of this trial.
- Qualitative data are to be presented as numbers, percentages, and 95% CI, and quantitative data are to be presented as means and standard deviations.
- Analyses of the primary, secondary, and other outcomes will be performed by calculating the 95% CI of the difference: mean incidence (no-hydrocortisone group) – mean incidence (hydrocortisone group).
- Non-inferiority is identified if the upper limit of the 95% CI is smaller than the margin of 10 percent points.

- The student's t-test will be used for comparisons of continuous variables and chi-squared test for categorical variables.
- A P value < 0.05 is considered statistically significant.

10. Randomization sequence

VAR00001	Random	Group	VAR00001	Random	Group	VAR00001	Random	Group
1	0.87	2	31	0.54	2	61	0.26	1
2	0.34	1	32	0.58	2	62	0.91	2
3	0.60	2	33	0.96	2	63	0.20	1
4	0.04	1	34	0.21	1	64	0.23	1
5	0.56	2	35	0.41	1	65	0.34	1
6	0.91	2	36	0.74	2	66	0.08	1
7	0.94	2	37	0.76	2	67	0.41	1
8	0.85	2	38	0.53	2	68	0.57	2
9	0.47	2	39	0.99	2	69	0.46	1
10	0.71	2	40	0.32	1	70	0.30	1
11	0.57	2	41	0.30	1	71	0.60	2
12	0.55	2	42	0.34	1	72	0.13	1
13	0.52	2	43	0.43	1	73	0.33	1
14	0.45	1	44	0.50	2	74	0.99	2
15	0.79	2	45	0.57	2	75	0.63	2
16	0.29	1	46	0.19	1	76	0.23	1
17	0.11	1	47	0.17	1	77	0.99	2
18	0.10	1	48	0.13	1	78	0.26	1
19	0.36	1	49	0.36	1	79	0.59	2
20	0.50	2	50	0.08	1	80	0.08	1
21	0.13	1	51	0.61	2	81	0.09	1
22	0.01	1	52	0.02	1	82	0.48	2
23	0.71	2	53	0.73	2	83	0.46	1
24	0.32	1	54	0.02	1	84	0.01	1
25	0.60	2	55	0.16	1	85	0.03	1
26	0.74	2	56	0.39	1	86	0.36	1
27	0.85	2	57	0.26	1	87	0.79	2
28	0.62	2	58	0.42	1	88	0.61	2
29	0.14	1	59	0.21	1	89	0.38	1
30	0.25	1	60	0.07	1	90	0.80	2

VAR00001	Random	Group	VAR00001	Random	Group	VAR00001	Random	Group
91	0.71	2	121	0.25	1	151	0.55	2
92	0.67	2	122	0.29	1	152	0.51	2
93	0.19	1	123	0.68	2	153	0.48	2
94	0.15	1	124	0.68	2	154	0.58	2
95	0.25	1	125	0.87	2	155	0.41	1
96	0.76	2	126	0.40	1	156	0.47	1
97	0.91	2	127	0.73	2	157	0.04	1
98	0.61	2	128	0.91	2	158	0.69	2
99	0.04	1	129	0.55	2	159	0.70	2
100	0.50	2	130	0.67	2	160	0.28	1
101	0.71	2	131	0.63	2	161	0.79	2
102	0.28	1	132	0.54	2	162	0.71	2
103	0.48	2	133	0.69	2	163	0.03	1
104	0.35	1	134	0.42	1	164	0.09	1
105	0.94	2	135	0.93	2	165	0.28	1
106	0.09	1	136	0.09	1	166	0.31	1
107	0.54	2	137	0.20	1	167	0.69	2
108	0.59	2	138	0.46	1	168	0.85	2
109	0.67	2	139	0.63	2	169	0.12	1
110	0.16	1	140	0.66	2	170	0.21	1
111	0.91	2	141	0.17	1	171	0.17	1
112	0.82	2	142	0.75	2	172	0.12	1
113	0.21	1	143	0.78	2	173	0.21	1
114	0.52	2	144	0.96	2	174	0.14	1
115	0.51	2	145	0.15	1	175	0.29	1
116	0.06	1	146	0.77	2	176	0.15	1
117	0.51	2	147	0.10	1	177	0.01	1
118	0.32	1	148	0.93	2	178	0.02	1
119	0.11	1	149	0.63	2	179	0.46	1
120	0.28	1	150	0.20	1	180	0.66	2

VAR00001	Random	Group	VAR00001	Random	Group	VAR00001	Random	Group
181	0.74	2	211	0.28	1	241	0.13	1
182	0.34	1	212	0.61	2	242	0.48	2
183	0.02	1	213	0.16	1	243	0.99	2
184	0.72	2	214	0.41	1	244	0.45	1
185	0.57	2	215	0.55	2	245	0.62	2
186	0.92	2	216	0.27	1	246	0.29	1
187	0.62	2	217	0.89	2	247	0.75	2
188	0.62	2	218	0.74	2	248	0.55	2
189	0.50	2	219	0.96	2	249	0.04	1
190	0.01	1	220	0.77	2	250	0.92	2
191	0.02	1	221	0.70	2	251	0.54	2
192	0.53	2	222	0.25	1	252	0.72	2
193	0.13	1	223	0.13	1	253	0.74	2
194	0.16	1	224	0.82	2	254	0.23	1
195	0.49	2	225	0.57	2	255	0.51	2
196	0.64	2	226	0.93	2	256	0.44	1
197	0.37	1	227	0.06	1	257	0.75	2
198	0.28	1	228	0.66	2	258	0.54	2
199	0.60	2	229	0.92	2	259	0.24	1
200	0.17	1	230	0.75	2	260	0.55	2
201	0.42	1	231	0.14	1	261	0.30	1
202	0.97	2	232	0.05	1	262	0.11	1
203	0.46	1	233	0.08	1	263	0.65	2
204	0.11	1	234	0.86	2	264	0.81	2
205	0.01	1	235	0.31	1	265	0.93	2
206	0.24	1	236	0.90	2	266	0.07	1
207	0.02	1	237	0.48	2	267	0.42	1
208	0.98	2	238	0.14	1	268	0.61	2
209	0.25	1	239	0.65	2	269	0.30	1
210	0.28	1	240	0.29	1	270	0.68	2

VAR00001	Random	Group	VAR00001	Random	Group	VAR00001	Random	Group
271	0.99	2	301	0.91	2	331	0.05	1
272	0.58	2	302	0.98	2	332	0.06	1
273	0.71	2	303	0.68	2	333	0.32	1
274	0.25	1	304	0.05	1	334	0.36	1
275	0.30	1	305	0.95	2	335	0.20	1
276	0.40	1	306	0.04	1	336	0.20	1
277	0.89	2	307	0.61	2	337	0.89	2
278	0.52	2	308	0.12	1	338	0.05	1
279	0.57	2	309	0.23	1	339	0.73	2
280	0.26	1	310	0.88	2	340	0.29	1
281	0.47	2	311	0.80	2	341	0.39	1
282	0.95	2	312	0.76	2	342	0.44	1
283	0.35	1	313	0.26	1	343	0.65	2
284	0.38	1	314	0.15	1	344	0.91	2
285	0.90	2	315	0.62	2	345	0.71	2
286	0.26	1	316	0.69	2	346	0.70	2
287	0.75	2	317	0.80	2	347	0.21	1
288	0.89	2	318	0.78	2	348	0.16	1
289	0.30	1	319	0.66	2	349	0.92	2
290	0.47	2	320	0.44	1	350	0.23	1
291	0.66	2	321	0.08	1	351	0.39	1
292	0.61	2	322	0.81	2	352	0.82	2
293	0.39	1	323	0.90	2	353	0.37	1
294	0.33	1	324	0.74	2	354	0.49	2
295	0.37	1	325	0.27	1	355	0.02	1
296	0.69	2	326	0.45	1	356	0.26	1
297	0.17	1	327	0.12	1	357	0.70	2
298	0.90	2	328	0.27	1	358	0.66	2
299	0.09	1	329	0.03	1	359	0.22	1
300	0.34	1	330	0.61	2	360	0.13	1

VAR00001	Random	Group	VAR00001	Random	Group	VAR00001	Random	Group
361	0.39	1	391	0.65	2	421	0.19	1
362	0.13	1	392	0.77	2	422	0.79	2
363	0.46	1	393	0.73	2	423	0.53	2
364	0.34	1	394	0.15	1	424	0.94	2
365	0.32	1	395	0.37	1	425	0.21	1
366	0.37	1	396	0.19	1	426	0.60	2
367	0.35	1	397	0.37	1	427	0.84	2
368	0.07	1	398	0.85	2	428	0.23	1
369	0.48	2	399	1.00	2	429	0.50	2
370	0.96	2	400	0.49	2	430	0.66	2
371	0.87	2	401	0.42	1	431	0.76	2
372	0.84	2	402	0.24	1	432	0.48	2
373	0.22	1	403	0.39	1	433	0.74	2
374	0.18	1	404	0.16	1	434	0.45	1
375	0.32	1	405	0.59	2	435	0.42	1
376	0.88	2	406	0.35	1	436	0.81	2
377	0.94	2	407	0.12	1			
378	0.32	1	408	0.19	1			
379	0.04	1	409	0.04	1			
380	0.88	2	410	0.56	2			
381	0.33	1	411	0.35	1			
382	0.70	2	412	0.65	2			
383	0.15	1	413	0.77	2			
384	1.00	2	414	0.48	2			
385	0.12	1	415	0.79	2			
386	0.91	2	416	0.43	1			
387	0.74	2	417	0.64	2			
388	0.27	1	418	0.11	1			
389	0.61	2	419	0.78	2			
390	0.55	2	420	0.82	2			

11. Informed Consent in English and Chinese

Non-use of Hydrocortisone vs. Conventional Hydrocortisone Replacement Therapy in Patients with Pituitary Adenomas with an Intact Hypothalamus-Pituitary-Adrenal Axis Function during the Perioperative Period: A Randomized Controlled Trial for Safety and Adverse Events

Version: 1.0 Date: Oct. 27, 2020

Principal Investigator: Bing Xing and Wei Lian
Phone Number: 86-10-69152530
Hospital Settings: Department of Neurosurgery, Peking Union Medical College Hospital, Chinese Academy of Medical Sciences and Peking Union Medical College Hospital, 1 Shuaifuyuan, Dongcheng District, Beijing 100730, China

Dear patient,
After a series of tests, we need to tell you that you are diagnosed with a pituitary adenoma (PA) and your hypothalamus-pituitary-adrenal (HPA) axis is intact. Here, we invite you to participate in an ongoing clinical trial titled "Non-use of Hydrocortisone vs. Conventional Hydrocortisone Replacement Therapy in Patients with Pituitary Adenomas with an Intact Hypothalamus-Pituitary-Adrenal Axis Function during the Perioperative Period: A Randomized Controlled Trial for Safety and Adverse Events". The trial has been approved by the institutional ethical committee at Peking Union Medical College Hospital (PUMCH).

Please read the following content carefully before you decide whether to participate in this study. It helps you understand the reasons why the trial is being conducted, the procedure and duration of the trial, and the possible benefits, risks, and discomforts that may come to you after taking part in the study. You can also discuss it with your family and friends, or ask your doctors to explain. All these can help you make a better decision. However, the choice is finally in your hands.

Background and Study Purpose

PA is the second most common primary brain tumor, the vast majority of which are benign. During the perioperative period, we have been routinely using hydrocortisone as a replacement therapy drug to prevent postoperative adrenal insufficiency (AI) and adrenal crisis, which can be even life-threatening. Recently, more and more studies from abroad have shown that withholding perioperative glucocorticoids do not significantly increase the risk of AI and adrenal crisis in patients with normal HPA axis function. In addition, it is known that glucocorticoid replacement therapy may bring a series of complications, such as Cushing's syndrome, susceptibility to infection, osteoporosis, and abnormal coagulation function.

Till now, however, there have been no randomized controlled trials (RCTs) with adequate sample sizes and feasible research protocols being published to guide the clinical practice for this group of patients.

PUMCH is the leader of the China Pituitary Disease Registry Center and China Pituitary Adenoma Specialist Council. The annual number of surgeries for PAs is 700-800. Thus, we have planned this trial taking advantage of our hospital to try to investigate whether withholding perioperative hydrocortisone supplementation was non-inferior to hydrocortisone replacement therapy in terms of the incidence of AI during perioperation in patients with an intact HPA-axis scheduled for pituitary adenomectomy.

Your participation will undoubtedly make an important contribution to obtaining such evidence and allow other patients to benefit from your contributions.

Inclusion Criteria:

- Patients with PAs who need surgical resection of the tumor via the transsphenoidal approach, whose HPA axis are also intact
- Patients of either gender aged from 18 years to 70 years

Exclusion Criteria:

- Patients with Cushing's disease
- Patients with PAs who have already developed secondary AI before surgery
- Patients with pituitary apoplexy or other acute pituitary conditions that need emergency surgery
- The postoperative pathology result indicates that the tumor is not a pituitary adenoma
- Patients that refuse to participate in the study or those who ask to quit after enrollment

What Should You Do in the Trial?

If you are an eligible patient and you are willing to participate in the study, you should first sign the informed consent. After you are admitted to the ward, we will randomly assign you to one of the following two treatment groups by computer randomization (similar to a coin toss or lottery) in a 1:1 ratio: 1) the hormone group; 2) No hormone group. Next, after preoperative tests, you will undergo transsphenoidal surgery to remove the tumor. We ask you to return to our outpatient clinic 3 months after surgery for a re-examination to further evaluate the safety and complications of hydrocortisone with or without it. Detailed information will be given to you by the nurses and residents who provide medical care during the perioperative period.

Potential Benefits

Your participation in the trial and the final result will provide an essential basis for the decision-making of whether or not to use steroids in patients with PAs with an intact HPA axis like you. In addition, you will receive upgraded support from our team after surgery for individualized instructions on the follow-up and rehabilitation: 1) you will receive a more detailed observation protocol and more intense follow-up to better guide your recovery. 2) we will set up a 7*24 online follow-up group to allow you to make timely consultations.

Potential Risks, Adverse Events, and Our Medical Responses

Adverse reactions that may be caused by the use of hydrocortisone include increased blood glucose level, electrolyte imbalance, systemic infection, promotion of hypercoagulable state and thrombosis, facial oil, acne, and osteoporosis. In this study, patients in the hormone group received hydrocortisone for about 17 days. Since patients with PAs in ours and other hospitals in China have been treated with the above protocol for more than 20 years, and no serious adverse reactions have been observed, the probability of adverse reactions in those receiving hydrocortisone is extremely low. For patients in the non-hormone group, according to literature data, the proportion of patients who may receive postoperative hormone replacement is about 5%-10%, all of which are at physiological doses. So, most patients who receive postoperative hormone replacement will not experience steroids-related adverse events. If severe steroid-related adverse reactions occur, we will immediately adopt the pre-specified treatment plans and protect your safety. Of note, the above adverse events will gradually improve and eventually disappear with the discontinuation of hydrocortisone.

For patients in the no-hydrocortisone group, one of the severe adverse events is postoperative AI or even adrenal crisis. In patients with PAs and an intact HPA axis, the literature reports that early postoperative AI occurs in less than 25% and sustains in <20% of patients. We have pre-defined the protocol of intense monitoring of the serum cortisol level and symptoms, and have prepared for the occurrence of AI. For patients who develop AI, we will give hormone replacement therapy in time, and the symptoms of AI will improve immediately after treatment before discharge. After discharge, we will invite you to join our WeChat group for patient follow-up, ask you about symptoms every other day, and provide follow-up instructions. If there is a suspected symptom, we will immediately guide you to seek medical support by telephone.

Another possible adverse reaction is the electrolyte disturbance such as hyponatremia, hypernatremia, or hypokalemia. We will require patients to review their blood electrolyte levels one week and two weeks after discharge and report online. We promise that if any abnormality is presented, we will contact the patient in time and provide medical guidance.

Fees and Compensation

All the drugs, tests, and operations for the patients enrolled in this trial are the same as patients that are not in. Thus, no additional medical costs are generated for trial patients. All the tests are necessary including pre-, intra-, and postoperative serum assays, radiological scans, hormone evaluations, deep vein ultrasound, and bone density assessment. All the drugs used during the perioperative period and all operations are the same with other patients with PAs.

Is the Medical Record Secret?

We will make every effort to protect the privacy of your personal medical information. Doctors will record your test results, treatment procedures, and follow-up instruction in the medical record. The medical records will all be stored in the medical record department at PUMCH. Only researchers of this trial and members of the ethical board are allowed to access your records. Please be notified that your personal information including but not limited to name,

phone, email, and address will not be presented in the database, and all public presentations will not include this information.

How to Get More Information about the Trial?

You are allowed to raise your question at any time on the online WeChat platform and will receive prompt answers for the doctors. If there is an emergency, feel free to dial the number of the doctor (17701220936). You have the right to ask questions about your rights or the associated risks. The number of the institutional ethical board is 69154494.

Participate or not?

Participation in this study or not is entirely up to your personal preference. You may refuse to participate, or withdraw at any time during the trial, without affecting your relationship with your doctors and without harming your interest. Your participation in this trial may be discontinued by your doctor at any time during the course for your benefit if a severe adverse event occurs. If you withdraw from this study for any reason, you may be ordered to undergo relevant laboratory tests and physical examinations if deemed clinically necessary.

What Should I Do Now?

It is time to decide whether or not to take part in this study. We would like to thank you for reading the above material. If you decide to take part in this program, please tell your doctor ASAP and he/she will arrange the following for you. Please keep this paper with you.

Declaration

- I have read the introduction of the trial and have had opportunities to discuss with doctors before decision-making. All my questions have been answered satisfactorily.
- I am aware of the possible risks and benefits of participating in this trial. I understand that participation in this study is voluntary, I acknowledge that I have spent sufficient time considering this, and I understand that I can always ask my doctor for more information.
- I can withdraw from this study at any time without discrimination or retaliation, and my medical treatment and rights will not be affected in any way.
- If I drop out in the middle of the study, especially due to hormone-related adverse events, I will tell my doctor about the changes in my condition and complete the corresponding physical examination and serum tests, which will be very beneficial to the whole study.
- If I need to take any other medication due to a change in my condition, I will seek the doctor's advice beforehand or tell the doctor truthfully afterward.
- I consent that the institutional Ethics Committee has the right to access my study data.
- I will get a signed and dated copy of the informed consent form.
- In the end, I decided to agree to participate in this study and pledged to do my best to follow my doctor's

orders.

Participant

Name (print)___________________Name (signature)_______________Date(m/d/y)___________________

Legal representative

Name (print)___________________Name (signature)_______________Date(m/d/y)___________________

Investigator

Name (print)___________________Name (signature)_______________Date(m/d/y)___________________

围手术期不应用氢化可的松 vs. 常规应用氢化可的松对 HPA 轴正常的垂体腺瘤患者安全性及并发症的前瞻性随机对照研究

知情同意书

版本号：1.0　　　　　版本日期：2020 年 10 月 27 日

研究负责人：幸兵 / 连伟/郭晓鹏
联系电话：010-69152530/17701220936
研究单位：中国医学科学院北京协和医院

亲爱的患者：

您好！经过一系列检验及检查，我们有义务告知您，您被确诊患有垂体腺瘤且下丘脑-垂体-肾上腺轴（HPA 轴）功能正常。我们诚挚邀请您参加一项临床研究："围手术期不应用氢化可的松 vs. 常规应用氢化可的松对 HPA 轴正常的垂体腺瘤患者安全性及并发症的前瞻性随机对照研究"。本研究方案已得到中国医学科学院北京协和医院伦理委员会审核，同意进行该项临床研究。

在您决定是否参加这项研究之前，请尽量仔细阅读以下内容。它可以帮助您了解为何要进行这项研究、研究的程序和期限、参加研究后可能给您带来的益处、风险和不适。如果您愿意的话，您也可以和家人、朋友一起讨论，或者请医生给予解释，帮助您做出决定。

一、 研究背景和研究目的

垂体腺瘤是中枢神经系统第三位常见的原发性肿瘤，绝大多数为良性。在围手术期，对于垂体腺瘤患者我们常规应用氢化可的松进行替代治疗，以预防术后肾上腺皮质功能低下及肾上腺危象的发生。近期，越来越多的来自国外的回顾性研究表明，对于 HPA 轴功能正常的垂体腺瘤患者，围手术期不应用糖皮质激素并不会显著增加肾上腺皮质功能低下及肾上腺危象的几率。众所周知，糖皮质激素替代治疗可能会带来一系列并发症，如医源性库欣综合征、易感染、骨质疏松、凝血功能异常等；但是截至目前，在该研究领域还没有一项样本量足够且研究方案较为完善的前瞻性随机对照研究（RCT）用以指导我国垂体腺瘤患者的临床诊疗实践。

北京协和医院神经外科是中国垂体腺瘤协作组前组长单位、中国垂体疾病注册中心，每年垂体腺瘤手术量约为 700-800 台。因此，本研究拟利用我科优势，开展前瞻性随机对照研究。我们的研究能够为垂体腺瘤患者是否必须在围手术期应用激素替代提供更多更有价值的证据。您的参与将为获得这样的证据做出极其重要的贡献，并使得其他患者从您的贡献中获益。

二、 哪些人不宜参加研究

本研究有严格的纳入标准和排除标准，凡不符合纳入标准的患者均不宜参加本研究；包括：垂体 ACTH 腺瘤患者、合并继发性肾上腺皮质功能低下的垂体腺瘤患者、垂体腺瘤卒中需急诊手术的患者、病理提示为非垂体腺瘤的其它鞍区病变患者、拒绝入组的患者等。

三、 如果参加研究将需要做什么？

首先，医生将根据您的病史、临床表现、检查检验结果等作出垂体腺瘤的临床诊断；之后会对您的 HPA 轴功能进行评估。若您是符合条件的纳入者，且您自愿参加研究，则可加入本研究并签署知情同意书。在您被收入病房后，我们将通过电脑随机分组的方式（类似于抛硬币或抽签）按照 1:1 比例将您分配至以下两个治疗组之一：1）应用激素组（对照组）；2）不应用激素组（试验组）。接下来您将接受经鼻蝶窦入路垂体腺瘤切除术。在您手术结束后 3 个月，我们要求您回我院门诊进行复查，对应用/不应用氢化可的松的安全性及并发症方面进行进一步评估。

四、 参加研究的可能受益

参加本研究，您并没有直接获益；但是本研究结果将为垂体腺瘤术后患者是否应用激素决策的制定提供依据。另外，在术后您将获得以下更为完善的诊治、随访及康复的支持。包括：1）更加完善的诊疗措施：包括更加细致和完善的对激素安全性及并发症的观察及随访，从而更好的指导评估。2）专门的随访、复查与咨询：本项目将设立随访微信群，让您得到及时、全面的病情咨询与监测，并对您在治疗后恢复及生活中有关垂体腺瘤的相关问题进行及时回答和处理。

五、 参加研究可能的风险、不良反应和应对措施

本研究干预措施为围手术期应用/不应用氢化可的松。长期大量应用氢化可的松可能造成的不良反应包括：血糖升高、电解质紊乱、诱发或加重感染、促进高凝状态及血栓形成、面部出油及痤疮增加、骨质疏松等。本研究中激素组患者共应用约 17 天氢化可的松（其中仅前 3 天为超短期/超生理剂量应用，后 14 天为生理剂量内应用）。由于我院乃至全国其他医院的垂体腺瘤患者近 20 年内均采用以上治疗方式，且未见严重不良反应发生，因此应用氢化可的松患者发生不良反应的几率极低；对于非激素组，结合文献数据，可能接受术后激素替代患者的比例约为 5%-10%，且均为生理剂量，因此绝大多数患者不会发生任何不良反应。如发生激素相关性不良反应，我们会立即采用相应的治疗方案。以上不良反应随氢化可的松停药将逐渐好转并最终消失。

对于不应用激素组的患者，一个可能的不良反应为术后突发肾上腺皮质功能低下。结合文献报道该发生率<10%；如果患者晨起血皮质醇水平降低且出现了乏力、恶心、心悸、高热等症状，则可诊断为肾上腺皮质功能低下。对于此类患者，在院内围手术期一经发现我们将及时予以激素替代治疗， 以上不良反应也将在激素替代后马上好转，之后我们会根据患者的血皮质醇复查结果及症状变化逐渐对氢化可的松进行减药并最终停药。在出院之后，我们建立相应的患者随访微信群，隔天询问患者的不适症状，如出现可疑的肾上腺皮质功能低下症状，我们将即刻通过电话的方式指导患者就诊。对于此类患者，另外一个可能的不良反应为电解质紊乱如低钠血症或低钾血症，我们将要求患者出院后在术后一周和两周分别复查血电解质水平并通过微信群的方式向我们反馈，如果发现有异常我们将及时联系患者并指导相关的诊治。

六、 有关费用和赔偿

本研究中入组患者所有用药、检验/检查、手术操作等项目收费与非入组患者的收费基本一致，因此并不增加受试者的任何医疗费用。1）检验检查方面：术前、围手术期、术后的血液学常规检查、垂体相关激素检查、下肢静脉超声检查及骨密度等检查均为垂体腺瘤患者诊疗及随访过程中的必要检查。2）用药、手术及随访方面：本研究中纳入的患者均接受经鼻蝶窦入路垂体腺瘤切除术及常规术后支持药物治疗，并要求术后 3 个月返院复查，与非入组患者诊疗方案完全一致。若您发生药物不良反应，研究者会按照临床常规进行处理。

七、 个人信息是保密的吗？

您的医疗记录（病历、化验单、检查结果等）将完整地保存在我院病案科。医生会将化验及其他检查结果记录在您的病历上。研究者及伦理委员会将被允许查阅您的医疗记录。您的所有个人信息，包括姓名、电话、电子邮件、住址等，均不会出现在本研究的电子数据库中，任何有关本研究结果的公开报告将不会披露您的个人身份及信息。我们将在法律允许范围内，尽一切努力保护您的个人医疗资料的隐私。

八、 怎样获得更多的信息？

您可以在任何时间提出有关本研究的任何问题，并得到相应解答。咨询电话（北京协和医院神经外科郭晓鹏医生）： 17701220936 。 您有权就有关您的权利或相关风险等问题进行咨询，咨询电话（北京协和医院伦理审查委员会）：69154494。

九、 可以自愿选择参加研究和中途退出研究

是否参加研究完全取决于您的意愿。您可以拒绝参加此项研究，或在研究过程中的任何时间退出本研究，这都不会影响您和医生间的关系，也不会对您的医疗或其他方面利益造成损失。

出于对您的最大利益考虑，医生或研究者可能在研究过程中随时中止您继续参加本研究。

如果您因为任何原因从本研究中退出，如果医生认为临床需要，您可能被要求进行相关实验室检查和体格检查。

十、 现在该做什么？

是否参加本项研究由您自己（和您家人）决定。

在您做出参加研究决定前，请尽可能向您的医生询问有关问题。

感谢您阅读以上材料。如果您决定参加本项目，请告诉您的医生，他/她会为您安排一切有关研究的事务。请您保留这份资料。

同意声明

我已经阅读了上述有关本研究的介绍，并且有机会就此项研究与医生讨论并提出有关问题。我提出的所有问题都得到了满意的答复。

我知道参加本研究可能产生的风险和受益。我知晓参加本研究是自愿的，我确认已有充足的时间对此进行考虑，而且明白：

- **我可以随时向医生咨询更多的信息。**
- **我可以随时退出本研究，而不会受到歧视或报复，医疗待遇与权益不会受到任何影响**

我同样清楚，如果我中途退出研究，特别是由于激素应用的原因使我退出研究时，我会将我的病情变化告诉医生，完成相应的体格检查和理化检查，这将对整个研究十分有利。

如果因病情变化我需要采取任何其他药物治疗，我会事先征求医生的意见，或在事后如实告诉医生。

我同意伦理委员会或申办者代表查阅我的研究资料。

我将获得一份经过签名并注明日期的知情同意书副本。

最后，我决定同意参加本项研究，并保证尽量遵从医嘱。

患者姓名（正楷）____________ 患者签字____________ 日期______________.

如患者不具备独立行为能力，则由法定代理人（监护人）签注，见下：

法定代理人姓名（正楷）__________ 法定代理人签字__________ 日期____________.

我确定已向患者解释了本研究的详细情况，并给其一份签署过的知情同意书副本。

研究者姓名（正楷）__________ 研究者签字__________ 日期____________.

Final Protocol and Statistical Analysis Plan

12. Brief Study Description

This is a single-center, parallel-group, non-inferiority, randomized, controlled trial. The investigators from Peking Union Medical College Hospital (PUMCH), Beijing, China, hypothesize that withholding hydrocortisone replacement therapy during the perioperative period in patients with pituitary adenomas (PAs) whose hypothalamus pituitary adrenal (HPA) axis function are intact are safe as compared with the conventional hydrocortisone replacement therapy.

This trial was approved by the Institutional Review Board at Peking Union Medical College Hospital (PUMCH, no. ZS-2608), and was registered with the *clinicaltrials.gov* database (no. NCT04621565).

13. Study Design

Study Type: Interventional (Clinical Trial)

Estimated Enrollment: 436 participants

Allocation: Randomized

Intervention Model: Parallel Assignment

Intervention Model Description: Participants are assigned to one of two groups, in a 1:1 ratio, to use hydrocortisone or not to use it during peri-operation of pituitary surgery.

Masking: Triple (Participant, Investigator, Outcomes Assessor)

Masking Description: Participants, investigators, and outcome assessors are all prevented from having knowledge of the interventions assigned to individual participants. Care providers (nurse and resident) know the individualized interventions.

14. Background Description

PAs account for approximately 17% of primary brain histologies and 90% of sellar lesions in adults, with an annual incidence of 4.36 per 100000 population in the United States.[1-3] Apart from hypersecretion symptoms, e.g. Cushing's syndrome and acromegaly, PAs can cause hypopituitarism by compressing the surrounding pituitary gland and thus increasing the pressure of sella.[4] Hypopituitarism can also be a surgical consequence due to the mechanical and vascular injury to the pituitary gland during adenomectomy.[4,5]

Adrenal insufficiency (AI) or the life-threatening adrenal crisis, presenting as dysfunction of the HPA axis, is one of the most lethal subtypes of hypopituitarism, leading to lethargy, fever, vomiting, tachycardia, low blood pressure, circulatory failure, and even death.[6] Since two cases were reported in the 1950s who died of acute postoperative AI because of withdrawal of glucocorticoids, stress-dose glucocorticoid replacement therapy has been recommended as the standard of care during perioperation of major surgeries, especially for the patients receiving continuous steroids replacement therapy.[7,8]

Perioperative glucocorticoid supplementation has long been a routine practice for patients with PAs.[9-12] There is consensus that patients with insufficient baseline HPA-axis function or low serum cortisol levels after surgery for Cushing's disease need steroids supplementation.[13,14] However, according to the recent non-randomized studies, for those with an intact preoperative HPA-axis, which accounts for the majority of patients with PAs, withholding perioperative steroids replacement therapy might be safe and not lead to a higher risk of postoperative AI and could avoid steroids-related adverse events.[10,11,15]

Two recent randomized trials suggested that perioperative steroids would be safely withheld under intensive monitoring of postoperative serum cortisol in patients with an intact HPA axis.[16,17]

However, because of the observative nature of non-randomized studies and the exceedingly small sample sizes (43 and 40 cases) that were not sufficient to calculate the incidence of postoperative AI and the improper types of glucocorticoid used (dexamethasone and prednisone) in the two clinical trials, a well-designed randomized trial with adequate sample size using the physiological type of steroids (hydrocortisone) is warranted to address this long-lasting but unsolved clinical concern.[18]

We aim to conduct this randomized trial to investigate whether the new protocol of withholding perioperative hydrocortisone supplementation is non-inferior to hydrocortisone replacement therapy in terms of the incidence of acute AI during perioperation in patients with an intact HPA-axis scheduled for pituitary adenomectomy. We hypothesize that the new treatment protocol is non-inferior to the conventional regimen, with an ultimate goal to minimize unnecessary steroid overuse. In addition, we evaluated the incidence of AI in the 3rd postoperative month, the prognostic factors for events, the incidences of adverse events during 3 months after surgery, and the pattern of postoperative HPA axis function changes without hydrocortisone supplementation.

References

1. Ostrom QT, Cioffi G, Waite K, et al. CBTRUS Statistical Report: Primary Brain and Other Central Nervous System Tumors Diagnosed in the United States in 2014-2018. Neuro Oncol 2021;23(12 Suppl 2):iii1-iii105. DOI: 10.1093/neuonc/noab200.
2. Emanuelli E, Zanotti C, Munari S, et al. Sellar and parasellar lesions: multidisciplinary management. Acta Otorhinolaryngol Ital 2021;41(Suppl. 1):S30-S41. DOI: 10.14639/0392-100X-suppl.1-41-2021-03.
3. Abele TA, Yetkin ZF, Raisanen JM, et al. Non-pituitary origin sellar tumours mimicking pituitary macroadenomas. Clin Radiol 2012;67(8):821-7. DOI: 10.1016/j.crad.2012.01.001.
4. Higham CE, Johannsson G, Shalet SM. Hypopituitarism. Lancet 2016;388(10058):2403-2415. DOI: 10.1016/S0140-6736(16)30053-8.
5. Molitch ME. Diagnosis and Treatment of Pituitary Adenomas: A Review. JAMA 2017;317(5):516-524. DOI: 10.1001/jama.2016.19699.
6. Husebye ES, Pearce SH, Krone NP, et al. Adrenal insufficiency. Lancet 2021;397(10274):613-629. DOI: 10.1016/S0140-6736(21)00136-7.
7. Fraser CG, Preuss FS, Bigford WD. Adrenal atrophy and irreversible shock associated with cortisone therapy. J Am Med Assoc 1952;149(17):1542-3. DOI: 10.1001/jama.1952.72930340001009.
8. Lewis L, Robinson RF, Yee J, et al. Fatal adrenal cortical insufficiency precipitated by surgery during prolonged continuous cortisone treatment. Ann Intern Med 1953;39(1):116-26. DOI: 10.7326/0003-4819-39-1-116.
9. Yeh PJ, Chen JW. Pituitary tumors: surgical and medical management. Surg Oncol 1997;6(2):67-92. DOI: 10.1016/s0960-7404(97)00008-x.
10. Borg H, Siesjo P, Kahlon B, et al. Perioperative serum cortisol levels in ACTH sufficient and ACTH deficient patients during transsphenoidal surgery of pituitary adenoma. Endocrine 2018;62(1):83-89. DOI: 10.1007/s12020-018-1655-8.
11. Tohti M, Li J, Zhou Y, et al. Is peri-operative steroid replacement therapy necessary for the pituitary adenomas treated with surgery? A systematic review and meta analysis. PLoS One 2015;10(3):e0119621. DOI: 10.1371/journal.pone.0119621.
12. Salem M, Tainsh RE, Jr., Bromberg J, et al. Perioperative glucocorticoid coverage. A reassessment 42 years after emergence of a problem. Ann Surg 1994;219(4):416-25. DOI: 10.1097/00000658-199404000-00013.
13. Inder WJ, Hunt PJ. Glucocorticoid replacement in pituitary surgery: guidelines for perioperative assessment and management. J Clin Endocrinol Metab 2002;87(6):2745-50. DOI: 10.1210/jcem.87.6.8547.
14. He X, Findling JW, Auchus RJ. Glucocorticoid Withdrawal Syndrome following treatment of endogenous Cushing Syndrome. Pituitary 2022. DOI: 10.1007/s11102-022-01218-y.
15. Hattori Y, Tahara S, Aso S, et al. Prophylactic steroid administration and complications after transsphenoidal pituitary surgery: a nationwide inpatient database study in Japan. Br J Anaesth 2021;127(2):e41-e43. DOI: 10.1016/j.bja.2021.04.006.
16. Lee HC, Yoon HK, Kim JH, et al. Comparison of intraoperative cortisol levels after preoperative hydrocortisone administration versus placebo in patients without adrenal insufficiency undergoing endoscopic transsphenoidal removal of nonfunctioning pituitary adenomas: a double-blind randomized trial. J Neurosurg 2020:1-9. DOI: 10.3171/2019.11.JNS192381.
17. Sterl K, Thompson B, Goss CW, et al. Withholding Perioperative Steroids in Patients Undergoing Transsphenoidal Resection for Pituitary Disease: Randomized Prospective Clinical Trial to Assess Safety. Neurosurgery 2019;85(2):E226-E232. DOI: 10.1093/neuros/nyy479.
18. Qiao N. Letter: Withholding Perioperative Steroids in Patients Undergoing Transsphenoidal Resection for Pituitary Disease: Randomized Prospective Clinical Trial to Assess Safety. Neurosurgery 2019;85(1):E161. DOI: 10.1093/neuros/nyz104.

15. Arms and Interventions

Arm 1:

Experimental: No-hydrocortisone group (Patients receive no perioperative hydrocortisone)

Intervention/treatment: No hydrocortisone or other steroids are given before, during, and after the surgery. These patients are given 100 ml of normal saline intravenously instead of hydrocortisone sodium succinate, which needs to be dissolved in 100 ml of normal saline in hydrocortisone group, followed by placebo tablets with a similar appearance to the hydrocortisone tablets. If a patient develops postoperative AI, he/she needs to receive hydrocortisone treatment (hydrocortisone sodium succinate, 100mg, iv., every 12 hours [q12h] for 2 days, and subsequent hydrocortisone tablets, 20mg, po., bid for 1 month). After these treatments, patient symptoms and serum cortisol levels are evaluated every week. The taper program should be started in patients with a normal morning serum cortisol level.

Arm 2:

Active Comparator: Hydrocortisone group (Patients receive perioperative hydrocortisone)

Intervention/treatment: Hydrocortisone sodium succinate dissolved in 100 ml of normal saline (0.9%) is given intravenously (iv.) on the day of operation (OD, 100 mg at 8 am and 100 mg at 8 pm), POD1 (100 mg at 8 am and 50 mg at 8 pm), and POD2 (25 mg at 8 am). Hydrocortisone tablets were subsequently given per mouth (po.) from the afternoon of POD2 (20 mg, twice a day [bid] for a week, and 20 mg once a day [qd] for another week) and were stopped at the start of the third postoperative week. If a patient develops postoperative AI, he/she needs to stop his/her original protocol and receive the modified hydrocortisone treatment regimen (hydrocortisone sodium succinate, 100mg, iv., every 12 hours [q12h] for 2 days, and subsequent hydrocortisone tablets, 20mg, po., bid for 1 month). After these treatments, patient symptoms and serum cortisol levels are evaluated every week. The taper program should be started in patients with a normal morning serum cortisol level.

16. Outcome Measures

1) Primary outcome

- Incidence of newly-onset **acute AI** [**During the perioperative period**]

Adrenal insufficiency: Lower-than-normal morning serum cortisol level (< 5 μg/dl) at 8 a.m., plus at least one of the AI-related symptoms.

The perioperative period: This period as defined in this trial includes the preoperative period in the ward, the day of operation, and the 1st and 2nd day after surgery.

2) Secondary outcome

- Incidence of newly-onset **late AI** [**From POD3 to the 3rd postoperative month**]

Adrenal insufficiency: Lower-than-normal morning serum cortisol level (< 5 μg/dl) at 8 a.m., plus at least one of the AI-related symptoms.

3) Other Outcome Measures:

- Rate of newly-developed **diabetes mellitus** [During the first 3 postoperative months]

  Diabetes mellitus: A fasting plasma glucose level ≥ 126 mg/dl (7.0 mmol/l) or the 2-h plasma glucose value ≥ 200 mg/dL (11.1 mmol/L) after the oral glucose tolerance test.

- Rate of newly-developed **diabetes insipidus** [During the first 3 postoperative months]

  Diabetes insipidus: Urine volume > 300 ml/h for more than 3 consecutive hours, urine specific gravity < 1.005, and serum sodium > 145 mmol/l.

- Rate of **electrolyte disturbance** [During the first 3 postoperative month]

Electrolyte disturbance is defined as lower-than- or higher-than-normal levels of serum sodium (135-145 mmol/l), potassium (3.5-5.5 mmol/l), and calcium (2.13-2.70 mmol/l).

- Rate of **deep venous thrombosis** [During the first 3 postoperative months]

Deep venous thrombosis is detected by the ultrasound.

- Rate of **decreased bone density** [During the first 3 postoperative months]

Decreased bone density is detected by the dual-energy x-ray absorptiometry.

17. Inclusion and Exclusion Criteria

Inclusion Criteria:

- Patients with PAs that need transsphenoidal surgery, whose HPA axis are intact
- Patients of either gender aged from 18 years to 70 years

Exclusion Criteria:

- Patients with Cushing's disease
- Patients with PAs who have AI before surgery
- Patients with pituitary apoplexy or other acute pituitary conditions that need emergency surgery
- The postoperative pathology result indicates that the tumor is not a PA
- Patients that refuse to participate or those who ask to quit after enrollment
- Patients who needed continuous glucocorticoid replacement therapy due to other diseases
- Patients who were pregnant

Patients Flow

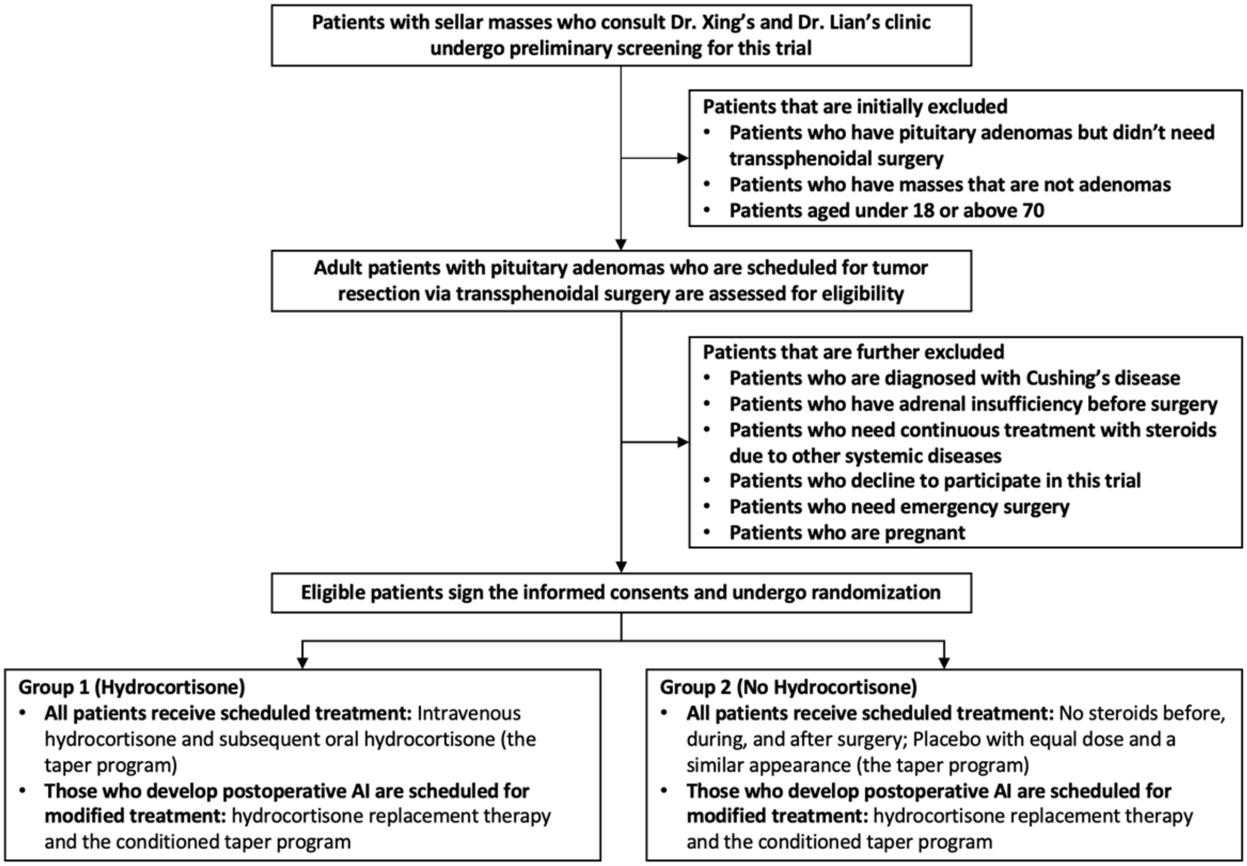

18. Sample Size Estimation

- We design the study as a non-inferiority trial to address the clinical question of whether the no-hydrocortisone protocol is non-inferior to the traditional regimen regarding the incidences of new-onset acute AI during perioperation after surgery for PAs in patients with an intact HPA-axis.
- The sample size is calculated based on the between-group comparison of the primary outcome (the incidences of new-onset AI during perioperation) to detect a 10% difference on the incidence of AI (as indicated by the 95% CI) during perioperation of pituitary surgery. The non-inferiority margin (δ) is thus set as 0.1.
- The calculation formula for qualitative data in non-inferiority trials ($N=2\times[U\alpha+U\beta]^2\times P[1-P]/\delta^2$) is used, with α set at 0.05 and β at 0.2.
- According to the published literature (DOI: 10.3171/2019.11.JNS192381; DOI: 10.1093/neuros/nyy479) and our preliminary observation, we set σ as 0.2.
- Assuming a 10% loss to follow-up, the sample size is finally determined as 218 in each trial group and 436 in total.

19. Randomization, Masking, and Data Collection

- Patients are to be randomly assigned, in a 1:1 ratio, to undergo perioperative administration of hydrocortisone or not to undergo such administration.
- The randomization sequence will be generated before patient recruitment using SPSS Statistics software with a fixed model.
- The blinded assignment will be performed.
- The study staff who collected trial data and those who assess the outcomes, the investigators, and study participants and their families are all blinded to group assignment before unblinding.
- The executive nurses and neurosurgical residents who allocated drugs or placeboes know the treatment assignment and keep secret.
- Data collection: Serum cortisol level is evaluated at baseline (8 am), on the OD (three timings: after anesthesia induction, OD1; after nasal mucosa incision, OD2; and after tumor removal, OD3), POD1 (8 am)

and POD2 (8 am), and in the 3rd postoperative month (POM3, 8 am). Serum ACTH level is evaluated before surgery (8 am, baseline), on POD1 (8 am) and POD2 (8 am), and in POM3 (8 am).

20. Statistical Analysis Plan

- This is a single-center, parallel-group, non-inferiority, randomized, controlled trial.
- We follow the CONSORT guideline for reporting the result of this trial.
- Qualitative data are to be presented as numbers, percentages, and 95% CI, and quantitative data are to be presented as means and standard deviations.
- Analyses of the primary, secondary, and other outcomes will be performed by calculating the 95% CI of the difference: mean incidence (no-hydrocortisone group) – mean incidence (hydrocortisone group).
- Non-inferiority is identified if the upper limit of the 95% CI is smaller than the margin of 10 percent points.
- We assess superiority for the outcomes in which the non-inferiority is achieved.
- Post hoc subgroup analyses and prognostic analyses will be performed for the primary and secondary outcomes.
- We use the receiver operator characteristic (ROC) curve to detect the cut-off value of baseline serum cortisol in predicting early and late postoperative AI.
- The student's t-test will be used for comparisons of continuous variables and chi-squared test for categorical variables.
- A P value < 0.05 is considered statistically significant.

21. Randomization sequence

VAR00001	Random	Group	VAR00001	Random	Group	VAR00001	Random	Group
1	0.87	2	31	0.54	2	61	0.26	1
2	0.34	1	32	0.58	2	62	0.91	2
3	0.60	2	33	0.96	2	63	0.20	1
4	0.04	1	34	0.21	1	64	0.23	1
5	0.56	2	35	0.41	1	65	0.34	1
6	0.91	2	36	0.74	2	66	0.08	1
7	0.94	2	37	0.76	2	67	0.41	1
8	0.85	2	38	0.53	2	68	0.57	2
9	0.47	2	39	0.99	2	69	0.46	1
10	0.71	2	40	0.32	1	70	0.30	1
11	0.57	2	41	0.30	1	71	0.60	2
12	0.55	2	42	0.34	1	72	0.13	1
13	0.52	2	43	0.43	1	73	0.33	1
14	0.45	1	44	0.50	2	74	0.99	2
15	0.79	2	45	0.57	2	75	0.63	2
16	0.29	1	46	0.19	1	76	0.23	1
17	0.11	1	47	0.17	1	77	0.99	2
18	0.10	1	48	0.13	1	78	0.26	1
19	0.36	1	49	0.36	1	79	0.59	2
20	0.50	2	50	0.08	1	80	0.08	1
21	0.13	1	51	0.61	2	81	0.09	1
22	0.01	1	52	0.02	1	82	0.48	2
23	0.71	2	53	0.73	2	83	0.46	1
24	0.32	1	54	0.02	1	84	0.01	1
25	0.60	2	55	0.16	1	85	0.03	1
26	0.74	2	56	0.39	1	86	0.36	1
27	0.85	2	57	0.26	1	87	0.79	2
28	0.62	2	58	0.42	1	88	0.61	2
29	0.14	1	59	0.21	1	89	0.38	1
30	0.25	1	60	0.07	1	90	0.80	2

VAR00001	Random	Group	VAR00001	Random	Group	VAR00001	Random	Group
91	0.71	2	121	0.25	1	151	0.55	2
92	0.67	2	122	0.29	1	152	0.51	2
93	0.19	1	123	0.68	2	153	0.48	2
94	0.15	1	124	0.68	2	154	0.58	2
95	0.25	1	125	0.87	2	155	0.41	1
96	0.76	2	126	0.40	1	156	0.47	1
97	0.91	2	127	0.73	2	157	0.04	1
98	0.61	2	128	0.91	2	158	0.69	2
99	0.04	1	129	0.55	2	159	0.70	2
100	0.50	2	130	0.67	2	160	0.28	1
101	0.71	2	131	0.63	2	161	0.79	2
102	0.28	1	132	0.54	2	162	0.71	2
103	0.48	2	133	0.69	2	163	0.03	1
104	0.35	1	134	0.42	1	164	0.09	1
105	0.94	2	135	0.93	2	165	0.28	1
106	0.09	1	136	0.09	1	166	0.31	1
107	0.54	2	137	0.20	1	167	0.69	2
108	0.59	2	138	0.46	1	168	0.85	2
109	0.67	2	139	0.63	2	169	0.12	1
110	0.16	1	140	0.66	2	170	0.21	1
111	0.91	2	141	0.17	1	171	0.17	1
112	0.82	2	142	0.75	2	172	0.12	1
113	0.21	1	143	0.78	2	173	0.21	1
114	0.52	2	144	0.96	2	174	0.14	1
115	0.51	2	145	0.15	1	175	0.29	1
116	0.06	1	146	0.77	2	176	0.15	1
117	0.51	2	147	0.10	1	177	0.01	1
118	0.32	1	148	0.93	2	178	0.02	1
119	0.11	1	149	0.63	2	179	0.46	1
120	0.28	1	150	0.20	1	180	0.66	2

VAR00001	Random	Group	VAR00001	Random	Group	VAR00001	Random	Group
181	0.74	2	211	0.28	1	241	0.13	1
182	0.34	1	212	0.61	2	242	0.48	2
183	0.02	1	213	0.16	1	243	0.99	2
184	0.72	2	214	0.41	1	244	0.45	1
185	0.57	2	215	0.55	2	245	0.62	2
186	0.92	2	216	0.27	1	246	0.29	1
187	0.62	2	217	0.89	2	247	0.75	2
188	0.62	2	218	0.74	2	248	0.55	2
189	0.50	2	219	0.96	2	249	0.04	1
190	0.01	1	220	0.77	2	250	0.92	2
191	0.02	1	221	0.70	2	251	0.54	2
192	0.53	2	222	0.25	1	252	0.72	2
193	0.13	1	223	0.13	1	253	0.74	2
194	0.16	1	224	0.82	2	254	0.23	1
195	0.49	2	225	0.57	2	255	0.51	2
196	0.64	2	226	0.93	2	256	0.44	1
197	0.37	1	227	0.06	1	257	0.75	2
198	0.28	1	228	0.66	2	258	0.54	2
199	0.60	2	229	0.92	2	259	0.24	1
200	0.17	1	230	0.75	2	260	0.55	2
201	0.42	1	231	0.14	1	261	0.30	1
202	0.97	2	232	0.05	1	262	0.11	1
203	0.46	1	233	0.08	1	263	0.65	2
204	0.11	1	234	0.86	2	264	0.81	2
205	0.01	1	235	0.31	1	265	0.93	2
206	0.24	1	236	0.90	2	266	0.07	1
207	0.02	1	237	0.48	2	267	0.42	1
208	0.98	2	238	0.14	1	268	0.61	2
209	0.25	1	239	0.65	2	269	0.30	1
210	0.28	1	240	0.29	1	270	0.68	2

VAR00001	Random	Group	VAR00001	Random	Group	VAR00001	Random	Group
271	0.99	2	301	0.91	2	331	0.05	1
272	0.58	2	302	0.98	2	332	0.06	1
273	0.71	2	303	0.68	2	333	0.32	1
274	0.25	1	304	0.05	1	334	0.36	1
275	0.30	1	305	0.95	2	335	0.20	1
276	0.40	1	306	0.04	1	336	0.20	1
277	0.89	2	307	0.61	2	337	0.89	2
278	0.52	2	308	0.12	1	338	0.05	1
279	0.57	2	309	0.23	1	339	0.73	2
280	0.26	1	310	0.88	2	340	0.29	1
281	0.47	2	311	0.80	2	341	0.39	1
282	0.95	2	312	0.76	2	342	0.44	1
283	0.35	1	313	0.26	1	343	0.65	2
284	0.38	1	314	0.15	1	344	0.91	2
285	0.90	2	315	0.62	2	345	0.71	2
286	0.26	1	316	0.69	2	346	0.70	2
287	0.75	2	317	0.80	2	347	0.21	1
288	0.89	2	318	0.78	2	348	0.16	1
289	0.30	1	319	0.66	2	349	0.92	2
290	0.47	2	320	0.44	1	350	0.23	1
291	0.66	2	321	0.08	1	351	0.39	1
292	0.61	2	322	0.81	2	352	0.82	2
293	0.39	1	323	0.90	2	353	0.37	1
294	0.33	1	324	0.74	2	354	0.49	2
295	0.37	1	325	0.27	1	355	0.02	1
296	0.69	2	326	0.45	1	356	0.26	1
297	0.17	1	327	0.12	1	357	0.70	2
298	0.90	2	328	0.27	1	358	0.66	2
299	0.09	1	329	0.03	1	359	0.22	1
300	0.34	1	330	0.61	2	360	0.13	1

VAR00001	Random	Group	VAR00001	Random	Group	VAR00001	Random	Group
361	0.39	1	391	0.65	2	421	0.19	1
362	0.13	1	392	0.77	2	422	0.79	2
363	0.46	1	393	0.73	2	423	0.53	2
364	0.34	1	394	0.15	1	424	0.94	2
365	0.32	1	395	0.37	1	425	0.21	1
366	0.37	1	396	0.19	1	426	0.60	2
367	0.35	1	397	0.37	1	427	0.84	2
368	0.07	1	398	0.85	2	428	0.23	1
369	0.48	2	399	1.00	2	429	0.50	2
370	0.96	2	400	0.49	2	430	0.66	2
371	0.87	2	401	0.42	1	431	0.76	2
372	0.84	2	402	0.24	1	432	0.48	2
373	0.22	1	403	0.39	1	433	0.74	2
374	0.18	1	404	0.16	1	434	0.45	1
375	0.32	1	405	0.59	2	435	0.42	1
376	0.88	2	406	0.35	1	436	0.81	2
377	0.94	2	407	0.12	1			
378	0.32	1	408	0.19	1			
379	0.04	1	409	0.04	1			
380	0.88	2	410	0.56	2			
381	0.33	1	411	0.35	1			
382	0.70	2	412	0.65	2			
383	0.15	1	413	0.77	2			
384	1.00	2	414	0.48	2			
385	0.12	1	415	0.79	2			
386	0.91	2	416	0.43	1			
387	0.74	2	417	0.64	2			
388	0.27	1	418	0.11	1			
389	0.61	2	419	0.78	2			
390	0.55	2	420	0.82	2			

22. Informed Consent in English and Chinese

Non-use of Hydrocortisone vs. Conventional Hydrocortisone Replacement Therapy in Patients with Pituitary Adenomas with an Intact Hypothalamus-Pituitary-Adrenal Axis Function during the Perioperative Period: A Randomized Controlled Trial for Safety and Adverse Events

Version: 1.0 Date: Oct. 27, 2020

Principal Investigator: Bing Xing and Wei Lian
Phone Number: 86-10-69152530
Hospital Settings: Department of Neurosurgery, Peking Union Medical College Hospital, Chinese Academy of Medical Sciences and Peking Union Medical College Hospital, 1 Shuaifuyuan, Dongcheng District, Beijing 100730, China

Dear patient,
After a series of tests, we need to tell you that you are diagnosed with a pituitary adenoma (PA) and your hypothalamus-pituitary-adrenal (HPA) axis is intact. Here, we invite you to participate in an ongoing clinical trial titled "Non-use of Hydrocortisone vs. Conventional Hydrocortisone Replacement Therapy in Patients with Pituitary Adenomas with an Intact Hypothalamus-Pituitary-Adrenal Axis Function during the Perioperative Period: A Randomized Controlled Trial for Safety and Adverse Events". The trial has been approved by the institutional ethical committee at Peking Union Medical College Hospital (PUMCH).

Please read the following content carefully before you decide whether to participate in this study. It helps you understand the reasons why the trial is being conducted, the procedure and duration of the trial, and the possible benefits, risks, and discomforts that may come to you after taking part in the study. You can also discuss it with your family and friends, or ask your doctors to explain. All these can help you make a better decision. However, the choice is finally in your hands.

Background and Study Purpose

PA is the second most common primary brain tumor, the vast majority of which are benign. During the perioperative period, we have been routinely using hydrocortisone as a replacement therapy drug to prevent postoperative adrenal insufficiency (AI) and adrenal crisis, which can be even life-threatening. Recently, more and more studies from abroad have shown that withholding perioperative glucocorticoids do not significantly increase the risk of AI and adrenal crisis in patients with normal HPA axis function. In addition, it is known that glucocorticoid replacement therapy may bring a series of complications, such as Cushing's syndrome, susceptibility to infection, osteoporosis, and abnormal coagulation function.

Till now, however, there have been no randomized controlled trials (RCTs) with adequate sample sizes and feasible research protocols being published to guide the clinical practice for this group of patients.

PUMCH is the leader of the China Pituitary Disease Registry Center and China Pituitary Adenoma Specialist Council. The annual number of surgeries for PAs is 700-800. Thus, we have planned this trial taking advantage of our hospital to try to investigate whether withholding perioperative hydrocortisone supplementation was non-inferior to hydrocortisone replacement therapy in terms of the incidence of AI during perioperation in patients with an intact HPA-axis scheduled for pituitary adenomectomy.

Your participation will undoubtedly make an important contribution to obtaining such evidence and allow other patients to benefit from your contributions.

Inclusion Criteria:

- Patients with PAs who need surgical resection of the tumor via the transsphenoidal approach, whose HPA axis are also intact
- Patients of either gender aged from 18 years to 70 years

Exclusion Criteria:

- Patients with Cushing's disease
- Patients with PAs who have already developed secondary AI before surgery
- Patients with pituitary apoplexy or other acute pituitary conditions that need emergency surgery
- The postoperative pathology result indicates that the tumor is not a pituitary adenoma
- Patients that refuse to participate in the study or those who ask to quit after enrollment

What Should You Do in the Trial?

If you are an eligible patient and you are willing to participate in the study, you should first sign the informed consent. After you are admitted to the ward, we will randomly assign you to one of the following two treatment groups by computer randomization (similar to a coin toss or lottery) in a 1:1 ratio: 1) the hormone group; 2) No hormone group. Next, after preoperative tests, you will undergo transsphenoidal surgery to remove the tumor. We ask you to return to our outpatient clinic 3 months after surgery for a re-examination to further evaluate the safety and complications of hydrocortisone with or without it. Detailed information will be given to you by the nurses and residents who provide medical care during the perioperative period.

Potential Benefits

Your participation in the trial and the final result will provide an essential basis for the decision-making of whether or not to use steroids in patients with PAs with an intact HPA axis like you. In addition, you will receive upgraded support from our team after surgery for individualized instructions on the follow-up and rehabilitation: 1) you will receive a more detailed observation protocol and more intense follow-up to better guide your recovery. 2) we will set up a 7*24 online follow-up group to allow you to make timely consultations.

Potential Risks, Adverse Events, and Our Medical Responses

Adverse reactions that may be caused by the use of hydrocortisone include increased blood glucose level, electrolyte imbalance, systemic infection, promotion of hypercoagulable state and thrombosis, facial oil, acne, and osteoporosis. In this study, patients in the hormone group received hydrocortisone for about 17 days. Since patients with PAs in ours and other hospitals in China have been treated with the above protocol for more than 20 years, and no serious adverse reactions have been observed, the probability of adverse reactions in those receiving hydrocortisone is extremely low. For patients in the non-hormone group, according to literature data, the proportion of patients who may receive postoperative hormone replacement is about 5%-10%, all of which are at physiological doses. So, most patients who receive postoperative hormone replacement will not experience steroids-related adverse events. If severe steroid-related adverse reactions occur, we will immediately adopt the pre-specified treatment plans and protect your safety. Of note, the above adverse events will gradually improve and eventually disappear with the discontinuation of hydrocortisone.

For patients in the no-hydrocortisone group, one of the severe adverse events is postoperative AI or even adrenal crisis. In patients with PAs and an intact HPA axis, the literature reports that early postoperative AI occurs in less than 25% and sustains in <20% of patients. We have pre-defined the protocol of intense monitoring of the serum cortisol level and symptoms, and have prepared for the occurrence of AI. For patients who develop AI, we will give hormone replacement therapy in time, and the symptoms of AI will improve immediately after treatment before discharge. After discharge, we will invite you to join our WeChat group for patient follow-up, ask you about symptoms every other day, and provide follow-up instructions. If there is a suspected symptom, we will immediately guide you to seek medical support by telephone.

Another possible adverse reaction is the electrolyte disturbance such as hyponatremia, hypernatremia, or hypokalemia. We will require patients to review their blood electrolyte levels one week and two weeks after discharge and report online. We promise that if any abnormality is presented, we will contact the patient in time and provide medical guidance.

Fees and Compensation

All the drugs, tests, and operations for the patients enrolled in this trial are the same as patients that are not in. Thus, no additional medical costs are generated for trial patients. All the tests are necessary including pre-, intra-, and postoperative serum assays, radiological scans, hormone evaluations, deep vein ultrasound, and bone density assessment. All the drugs used during the perioperative period and all operations are the same with other patients with PAs.

Is the Medical Record Secret?

We will make every effort to protect the privacy of your personal medical information. Doctors will record your test results, treatment procedures, and follow-up instruction in the medical record. The medical records will all be stored in the medical record department at PUMCH. Only researchers of this trial and members of the ethical board are allowed to access your records. Please be notified that your personal information including but not limited to name,

phone, email, and address will not be presented in the database, and all public presentations will not include this information.

How to Get More Information about the Trial?

You are allowed to raise your question at any time on the online WeChat platform and will receive prompt answers for the doctors. If there is an emergency, feel free to dial the number of the doctor (17701220936). You have the right to ask questions about your rights or the associated risks. The number of the institutional ethical board is 69154494.

Participate or not?

Participation in this study or not is entirely up to your personal preference. You may refuse to participate, or withdraw at any time during the trial, without affecting your relationship with your doctors and without harming your interest. Your participation in this trial may be discontinued by your doctor at any time during the course for your benefit if a severe adverse event occurs. If you withdraw from this study for any reason, you may be ordered to undergo relevant laboratory tests and physical examinations if deemed clinically necessary.

What Should I Do Now?

It is time to decide whether or not to take part in this study. We would like to thank you for reading the above material. If you decide to take part in this program, please tell your doctor ASAP and he/she will arrange the following for you. Please keep this paper with you.

Declaration

- I have read the introduction of the trial and have had opportunities to discuss with doctors before decision-making. All my questions have been answered satisfactorily.
- I am aware of the possible risks and benefits of participating in this trial. I understand that participation in this study is voluntary, I acknowledge that I have spent sufficient time considering this, and I understand that I can always ask my doctor for more information.
- I can withdraw from this study at any time without discrimination or retaliation, and my medical treatment and rights will not be affected in any way.
- If I drop out in the middle of the study, especially due to hormone-related adverse events, I will tell my doctor about the changes in my condition and complete the corresponding physical examination and serum tests, which will be very beneficial to the whole study.
- If I need to take any other medication due to a change in my condition, I will seek the doctor's advice beforehand or tell the doctor truthfully afterward.
- I consent that the institutional Ethics Committee has the right to access my study data.
- I will get a signed and dated copy of the informed consent form.
- In the end, I decided to agree to participate in this study and pledged to do my best to follow my doctor's

orders.

Participant

Name (print)____________________Name (signature)________________Date(m/d/y)____________________

Legal representative

Name (print)____________________Name (signature)________________Date(m/d/y)____________________

Investigator

Name (print)____________________Name (signature)________________Date(m/d/y)____________________

围手术期不应用氢化可的松 vs. 常规应用氢化可的松对 HPA 轴正常的垂体腺瘤患者安全性及并发症的前瞻性随机对照研究

知情同意书

版本号：1.0　　　　版本日期：2020 年 10 月 27 日

研究负责人：幸兵 / 连伟/郭晓鹏
联系电话：010-69152530/17701220936
研究单位：中国医学科学院北京协和医院

亲爱的患者：

您好！经过一系列检验及检查，我们有义务告知您，您被确诊患有垂体腺瘤且下丘脑-垂体-肾上腺轴（HPA 轴）功能正常。我们诚挚邀请您参加一项临床研究："围手术期不应用氢化可的松 vs. 常规应用氢化可的松对 HPA 轴正常的垂体腺瘤患者安全性及并发症的前瞻性随机对照研究"。本研究方案已得到中国医学科学院北京协和医院伦理委员会审核，同意进行该项临床研究。

在您决定是否参加这项研究之前，请尽量仔细阅读以下内容。它可以帮助您了解为何要进行这项研究、研究的程序和期限、参加研究后可能给您带来的益处、风险和不适。如果您愿意的话，您也可以和家人、朋友一起讨论，或者请医生给予解释，帮助您做出决定。

十一、 研究背景和研究目的

垂体腺瘤是中枢神经系统第三位常见的原发性肿瘤，绝大多数为良性。在围手术期，对于垂体腺瘤患者我们常规应用氢化可的松进行替代治疗，以预防术后肾上腺皮质功能低下及肾上腺危象的发生。近期，越来越多的来自国外的回顾性研究表明，对于 HPA 轴功能正常的垂体腺瘤患者，围手术期不应用糖皮质激素并不会显著增加肾上腺皮质功能低下及肾上腺危象的几率。众所周知，糖皮质激素替代治疗可能会带来一系列并发症，如医源性库欣综合征、易感染、骨质疏松、凝血功能异常等；但是截至目前，在该研究领域还没有一项样本量足够且研究方案较为完善的前瞻性随机对照研究（RCT）用以指导我国垂体腺瘤患者的临床诊疗实践。

北京协和医院神经外科是中国垂体腺瘤协作组前组长单位、中国垂体疾病注册中心，每年垂体腺瘤手术量约为 700-800 台。因此，本研究拟利用我科优势，开展前瞻性随机对照研究。我们的研究能够为垂体腺瘤患者是否必须在围手术期应用激素替代提供更多更有价值的证据。您的参与将为获得这样的证据做出极其重要的贡献，并使得其他患者从您的贡献中获益。

十二、 哪些人不宜参加研究

本研究有严格的纳入标准和排除标准，凡不符合纳入标准的患者均不宜参加本研究；包括：垂体 ACTH 腺瘤患者、合并继发性肾上腺皮质功能低下的垂体腺瘤患者、垂体腺瘤卒中需急诊手术的患者、病理提示为非垂体腺瘤的其它鞍区病变患者、拒绝入组的患者等。

十三、 如果参加研究将需要做什么？

首先，医生将根据您的病史、临床表现、检查检验结果等作出垂体腺瘤的临床诊断；之后会对您的 HPA 轴功能进行评估。若您是符合条件的纳入者，且您自愿参加研究，则可加入本研究并签署知情同意书。在您被收入病房后，我们将通过电脑随机分组的方式（类似于抛硬币或抽签）按照 1:1 比例将您分配至以下两个治疗组之一：1）应用激素组（对照组）；2）不应用激素组（试验组）。接下来您将接受经鼻蝶窦入路垂体腺瘤切除术。在您手术结束后 3 个月，我们要求您回我院门诊进行复查，对应用/不应用氢化可的松的安全性及并发症方面进行进一步评估。

十四、 参加研究的可能受益

参加本研究，您并没有直接获益；但是本研究结果将为垂体腺瘤术后患者是否应用激素决策的制定提供依据。另外，在术后您将获得以下更为完善的诊治、随访及康复的支持。包括：1）更加完善的诊疗措施：包括更加细致和完善的对激素安全性及并发症的观察及随访，从而更好的指导评估。2）专门的随访、复查与咨询：本项目将设立随访微信群，让您得到及时、全面的病情咨询与监测，并对您在治疗后恢复及生活中有关垂体腺瘤的相关问题进行及时回答和处理。

十五、 参加研究可能的风险、不良反应和应对措施

本研究干预措施为围手术期应用/不应用氢化可的松。长期大量应用氢化可的松可能造成的不良反应包括：血糖升高、电解质紊乱、诱发或加重感染、促进高凝状态及血栓形成、面部出油及痤疮增加、骨质疏松等。本研究中激素组患者共应用约 17 天氢化可的松（其中仅前 3 天为超短期/超生理剂量应用，后 14 天为生理剂量内应用）。由于我院乃至全国其他医院的垂体腺瘤患者近 20 年内均采用以上治疗方式，且未见严重不良反应发生，因此应用氢化可的松患者发生不良反应的几率极低；对于非激素组，结合文献数据，可能接受术后激素替代患者的比例约为 5%-10%，且均为生理剂量，因此绝大多数患者不会发生任何不良反应。如发生激素相关性不良反应，我们会立即采用相应的治疗方案。以上不良反应随氢化可的松停药将逐渐好转并最终消失。

对于不应用激素组的患者，一个可能的不良反应为术后突发肾上腺皮质功能低下。结合文献报道该发生率<10%；如果患者晨起血皮质醇水平降低且出现了乏力、恶心、心悸、高热等症状，则可诊断为肾上腺皮质功能低下。对于此类患者，在院内围手术期一经发现我们将及时予以激素替代治疗， 以上不良反应也将在激素替代后马上好转，之后我们会根据患者的血皮质醇复查结果及症状变化逐渐对氢化可的松进行减药并最终停药。在出院之后，我们建立相应的患者随访微信群，隔天询问患者的不适症状，如出现可疑的肾上腺皮质功能低下症状，我们将即刻通过电话的方式指导患者就诊。对于此类患者，另外一个可能的不良反应为电解质紊乱如低钠血症或低钾血症，我们将要求患者出院后在术后一周和两周分别复查血电解质水平并通过微信群的方式向我们反馈，如果发现有异常我们将及时联系患者并指导相关的诊治。

十六、 有关费用和赔偿

本研究中入组患者所有用药、检验/检查、手术操作等项目收费与非入组患者的收费基本一致，因此并不增加受试者的任何医疗费用。1）检验检查方面：术前、围手术期、术后的血液学常规检查、垂体相关激素检查、下肢静脉超声检查及骨密度等检查均为垂体腺瘤患者诊疗及随访过程中的必要检查。2）用药、手术及随访方面：本研究中纳入的患者均接受经鼻蝶窦入路垂体腺瘤切除术及常规术后支持药物治疗，并要求术后 3 个月返院复查，与非入组患者诊疗方案完全一致。若您发生药物不良反应，研究者会按照临床常规进行处理。

十七、 个人信息是保密的吗？

您的医疗记录（病历、化验单、检查结果等）将完整地保存在我院病案科。医生会将化验及其他检查结果记录在您的病历上。研究者及伦理委员会将被允许查阅您的医疗记录。您的所有个人信息，包括姓名、电话、电子邮件、住址等，均不会出现在本研究的电子数据库中，任何有关本研究结果的公开报告将不会披露您的个人身份及信息。我们将在法律允许范围内，尽一切努力保护您的个人医疗资料的隐私。

十八、 怎样获得更多的信息？

您可以在任何时间提出有关本研究的任何问题，并得到相应解答。咨询电话（北京协和医院神经外科郭晓鹏医生）： 17701220936 。 您有权就有关您的权利或相关风险等问题进行咨询，咨询电话（北京协和医院伦理审查委员会）：69154494。

十九、 可以自愿选择参加研究和中途退出研究

是否参加研究完全取决于您的意愿。您可以拒绝参加此项研究，或在研究过程中的任何时间退出本研究，这都不会影响您和医生间的关系，也不会对您的医疗或其他方面利益造成损失。

出于对您的最大利益考虑，医生或研究者可能在研究过程中随时中止您继续参加本研究。

如果您因为任何原因从本研究中退出，如果医生认为临床需要，您可能被要求进行相关实验室检查和体格检查。

二十、 现在该做什么？

是否参加本项研究由您自己（和您家人）决定。

在您做出参加研究决定前，请尽可能向您的医生询问有关问题。

感谢您阅读以上材料。如果您决定参加本项目，请告诉您的医生，他/她会为您安排一切有关研究的事务。请您保留这份资料。

同意声明

我已经阅读了上述有关本研究的介绍，并且有机会就此项研究与医生讨论并提出有关问题。我提出的所有问题都得到了满意的答复。

我知道参加本研究可能产生的风险和受益。我知晓参加本研究是自愿的，我确认已有充足的时间对此进行考虑，而且明白：

- **我可以随时向医生咨询更多的信息。**
- **我可以随时退出本研究，而不会受到歧视或报复，医疗待遇与权益不会受到任何影响**

我同样清楚，如果我中途退出研究，特别是由于激素应用的原因使我退出研究时，我会将我的病情变化告诉医生，完成相应的体格检查和理化检查，这将对整个研究十分有利。

如果因病情变化我需要采取任何其他药物治疗，我会事先征求医生的意见，或在事后如实告诉医生。

我同意伦理委员会或申办者代表查阅我的研究资料。

我将获得一份经过签名并注明日期的知情同意书副本。

最后，我决定同意参加本项研究，并保证尽量遵从医嘱。

患者姓名（正楷）____________ 患者签字____________ 日期____________.

如患者不具备独立行为能力，则由法定代理人（监护人）签注，见下：

法定代理人姓名（正楷）__________ 法定代理人签字__________ 日期____________.

我确定已向患者解释了本研究的详细情况，并给其一份签署过的知情同意书副本。

研究者姓名（正楷）__________ 研究者签字__________ 日期____________.

Summary of Changes to the Protocol and the Statistical Analysis Plan

Topic	Initial Version	Final Version	Justification
Treatment and monitoring for the patients with new-onset postoperative AI	If a patient develops postoperative AI, he/she needs to receive hydrocortisone treatment (*20mg at 0800 and 20mg at 1600 for a month*) and then routinely check the level of morning serum cortisol to decide the optimal timing to start the hydrocortisone taper program.	If a patient develops postoperative AI, he/she needs to receive hydrocortisone treatment (*hydrocortisone sodium succinate, 100mg, iv., every 12 hours [q12h] for 2 days, and subsequent hydrocortisone tablets, 20mg, po., bid for 1 month*). After these treatments, patient symptoms and serum cortisol levels are evaluated every week. The taper program should be started in patients with a normal morning serum cortisol level and no symptoms.	The treatment protocol for patients with new-onset postoperative AI was changed to a combination of short-term intravenous hydrocortisone to achieve a rapid recovery of serum cortisol level and the following oral hydrocortisone tablets to maintain the status. In addition, we detailed the frequency of re-evaluation of morning serum cortisol level in patients who had completed the modified hydrocortisone treatment protocol.
Duration of taking hydrocortisone tablets in the hydrocortisone group	Hydrocortisone tablet (po.) is given starting from the afternoon of the postoperative day 2 (*20mg at 1600 and 0800, twice a day*) *to the end of the first postoperative week, and 20mg at 0800 during the second postoperative week.*	Hydrocortisone tablets are given per mouth (po.) from the afternoon of the postoperative day 2 (*20 mg, twice a day [bid] for a week, and 20 mg once a day [qd] for another week*) *and are stopped at the start of the third postoperative week.*	We slightly modified the duration of taking hydrocortisone tablets in the hydrocortisone group, in order to increase patient compliance (easy to memorize for patients) and to avoid possible wrong ways of taking medications.
Detailed description of the primary endpoint	The primary endpoint is new-onset AI *during the first 3 days after surgery.*<br>AI is defined as lower-than-normal serum cortisol level at 8 a.m., plus the following related symptoms, including serious fatigue, muscle weakness, decreased appetite, nausea, vomiting, diarrhea, low blood pressure, palpitation, and fever.	The primary endpoint is acute new-onset AI during the perioperative period. AI is defined as a lower morning serum cortisol level < 5 µg/dl at 8 a.m., plus at least one of the AI-related symptoms. The *perioperative period as defined in this trial includes the preoperative period in the ward, the day of operation, and the 1st and 2nd day after surgery.*	We specified the "lower-than-normal serum cortisol level at 8 a.m." as a morning cortisol level < 5 µg/dl. We defined the perioperative period in this trial as the period starting from the preoperative period to the 2nd day after surgery (during the first 3 days after surgery – day of operation [OD], POD1, POD2).
Definition of diabetes mellitus	A *random reading of blood sugar level more than 200 mg/dL (11.1 mmol/L)*, or a *reading after two hours (after OGTT) over 200 mg/dL (11.1 mmol/L).*	A *fasting plasma glucose level ≥ 126 mg/dl (7.0 mmol/l)* or the *2-h plasma glucose value ≥ 200 mg/dL (11.1 mmol/L)* after the oral glucose tolerance test.	We modified the definition of diabetes mellitus according to the current criteria (Classification and diagnosis of diabetes. Diabetes Care 2015;38 Suppl: S8-S16) and reevaluated the incidences accordingly as presented in the article.

Definition of diabetes insipidus	Urine volume > 300ml for more than 3h or > 6 liter per day, *specific gravity of urine <1.003,* and serum sodium levels > 145 mmol/L.	Urine volume > 300 ml/h for more than 3 consecutive hours, *urine specific gravity < 1.005,* and serum sodium > 145 mmol/l.	We modified the definition of diabetes insipidus according to the current criteria (Postoperative diabetes insipidus: how to define and grade this complication? Pituitary 2021;24(2):284-291) and reevaluated the incidences accordingly as presented in the article.
Definition of electrolyte disturbance	No detailed definition.	*Lower-than- or higher-than-normal levels of serum sodium (135-145 mmol/l), potassium (3.5-5.5 mmol/l), and calcium (2.13-2.70 mmol/l).*	We described the detailed subtypes of electrolyte disturbance and detailed their definitions.
Exclusion criteria	• Patients with Cushing's disease<br>• Patients with PAs who have AI before surgery<br>• Patients with pituitary apoplexy or other acute pituitary conditions that need emergency surgery<br>• The postoperative pathology result indicates that the tumor is not a PA<br>• Patients that refuse to participate or those who ask to quit after enrollment	• Patients with Cushing's disease<br>• Patients with PAs who have AI before surgery<br>• Patients with pituitary apoplexy or other acute pituitary conditions that need emergency surgery<br>• The postoperative pathology result indicates that the tumor is not a PA<br>• Patients that refuse to participate or those who ask to quit after enrollment<br>• *Patients who needed continuous glucocorticoid replacement therapy due to other diseases*<br>• *Patients who were pregnant*	We added two groups of patients who would not be allowed to be enrolled in this trial: patients who needed continuous glucocorticoid replacement therapy due to other diseases, and patients who were pregnant.

Statistical Analysis Plan	This is a single-center, triple-masking, parallel-group, non-inferiority, randomized, controlled trial. We follow the CONSORT guideline for reporting the result of this trial. Qualitative data are to be presented as numbers, percentages, and 95% CI, and quantitative data are to be presented as means and standard deviations. Analyses of the primary, secondary, and other outcomes will be performed by calculating the 95% CI of the difference: mean incidence (no-hydrocortisone group) – mean incidence (hydrocortisone group). Non-inferiority is identified if the upper limit of the 95% CI is smaller than the margin of 10 percent points. The student's t-test will be used for comparisons of continuous variables and chi-squared test for categorical variables. A P value < 0.05 is considered statistically significant.	This is a single-center, triple-masking, parallel-group, non-inferiority, randomized, controlled trial. We follow the CONSORT guideline for reporting the result of this trial. Qualitative data are to be presented as numbers, percentages, and 95% CI, and quantitative data are to be presented as means and standard deviations. Analyses of the primary, secondary, and other outcomes will be performed by calculating the 95% CI of the difference: mean incidence (no-hydrocortisone group) – mean incidence (hydrocortisone group). Non-inferiority is identified if the upper limit of the 95% CI is smaller than the margin of 10 percent points. *We assess superiority for the outcomes in which the non-inferiority is achieved. Post hoc subgroup analyses and prognostic analyses will be performed for the primary and secondary outcomes. We use the receiver operator characteristic (ROC) curve to detect the cut-off value of baseline serum cortisol in predicting early and late postoperative AI.* The student's t-test will be used for comparisons of continuous variables and chi-squared test for categorical variables. A P value < 0.05 is considered statistically significant.	Three terms were added in the final protocol of statistical analysis plan:<br>• We assess superiority for the outcomes in which the non-inferiority is achieved.<br>• Post hoc subgroup analyses and prognostic analyses will be performed for the primary and secondary outcomes.<br>• We use the receiver operator characteristic (ROC) curve to detect the cut-off value of baseline serum cortisol in predicting early and late postoperative AI.

The above changes to the Protocol and the Statistical Analysis Plan were not substantially different with the initial ones, but could significantly reduce patient risks and improve patient compliance.
