# Supplementary material for: Safety of Withholding Perioperative Hydrocortisone for Patients With Pituitary Adenomas With an Intact Hypothalamus-Pituitary-Adrenal Axis: A Randomized Clinical Trial
Source: JAMA Netw Open. 2022 Nov 16;5(11):e2242221. doi: 10.1001/jamanetworkopen.2022.42221 (PMC9669812; doi:10.1001/jamanetworkopen.2022.42221)
Supplement: Supplement 2. — eTable 1. List of the Patients With a Primary Event eTable 2. List of the Patients With a Secondary Event eTable 3. Subgroup Analyses of the Primary Outcome in the Intention-to-Treat Population eTable 4. Subgroup Analyses of the Secondary Outcome in the Intention-to-Treat Population eTable 5. Prognostic Factors Associated With a Primary Event in 436 Patients eTable 6. Prognostic Factors Associated With a Secondary Event in 436 Patients eTable 7. Adjusted Incidence Rate Difference and 95% CI for the Primary Outcome eFigure 1. ROC Curve for Preoperative Cortisol in Predicting AI Within 2 Days After Surgery eFigure 2. ROC Curve for Preoperative Cortisol in Predicting AI from POD3 to POM3 [file jamanetwopen-e2242221-s002.pdf]

Supplementary Online Content

Guo X, Zhang D, Pang H, et al; ZS-2608 Trial Team. Safety of withholding perioperative hydrocortisone for patients with pituitary adenomas with an intact hypothalamus-pituitary-adrenal axis: a randomized clinical trial. *JAMA Netw Open.* 2022;5(11):e2242221. doi:10.1001/jamanetworkopen.2022.42221

eTable 1. List of the Patients With a Primary Event

eTable 2. List of the Patients With a Secondary Event

eTable 3. Subgroup Analyses of the Primary Outcome in the Intention-to-Treat Population

eTable 4. Subgroup Analyses of the Secondary Outcome in the Intention-to-Treat Population

eTable 5. Prognostic Factors Associated With a Primary Event in 436 Patients

eTable 6. Prognostic Factors Associated With a Secondary Event in 436 Patients

eTable 7. Adjusted Incidence Rate Difference and 95% CI for the Primary Outcome

eFigure 1. ROC Curve for Preoperative Cortisol in Predicting AI Within 2 Days After Surgery

eFigure 2. ROC Curve for Preoperative Cortisol in Predicting AI from POD3 to POM3

This supplementary material has been provided by the authors to give readers additional information about their work.

© 2022 Guo X et al. *JAMA Network Open.*

eTable 1. List of the Patients With a Primary Event

Pt no.	Trial Group	Age	Sex	BMI	Medical history, 1=yes, 0=no	Tumor maximal diameter, mm	Macroadenoma, 1=yes, 0=no	Cavernous sinus invasion, 1=yes, 0=no	Tumor type	Hypopituitarism at baseline, 1=yes, 0=no	Gross total resection, 1=yes, 0=no
4	1	33	Male	32.6	0	13.0	1	0	GH	0	1
17	1	62	Male	27.7	1	13.0	1	0	NF	0	1
32	2	42	Male	21.8	1	11.0	1	1	GH	0	0
38	2	58	Male	23.7	1	39.0	1	1	NF	1	0
40	1	51	Male	27.6	1	30.0	1	0	NF	1	1
45	2	46	Female	24.7	1	9.4	0	0	GH	0	1
46	1	49	Female	23.5	1	12.0	1	0	GH	1	1
80	1	57	Male	26.4	1	26.0	1	1	NF	0	0
85	1	47	Male	25.6	1	21.0	1	0	NF	0	1
87	2	38	Male	27.4	1	35.0	1	0	NF	1	1
95	1	37	Female	22.6	0	20.0	1	0	NF	1	1
131	2	59	Male	24.0	1	21.0	1	0	NF	1	1
146	2	52	Female	26.7	1	31.0	1	1	NF	1	0
149	2	51	Male	26.6	1	24.0	1	0	NF	1	1
163	1	40	Female	32.4	1	35.0	1	1	NF	1	0
177	1	43	Male	30.6	1	14.0	1	0	NF	1	1
202	2	31	Female	22.6	0	17.5	1	0	NF	0	1
213	1	46	Female	26.1	1	16.0	1	1	GH	0	0
221	2	53	Male	20.8	0	16.0	1	0	NF	0	1
225	2	31	Male	26.3	0	14.0	1	0	PRL	1	1
229	2	26	Female	23.1	0	7.0	0	0	PRL	0	1
232	1	37	Female	20.3	0	9.0	0	0	PRL	0	1
236	2	50	Male	21.1	0	29.8	1	0	NF	0	1
239	2	32	Female	25.0	1	15.0	1	0	GH	1	1
247	2	41	Female	37.3	1	19.0	1	1	NF	0	0
253	2	33	Male	26.8	0	30.0	1	0	NF	1	1
274	1	35	Female	27.3	0	26.8	1	0	NF	0	1
278	2	45	Female	27.3	1	40.3	1	1	NF	0	0
287	2	31	Female	22.8	0	27.0	1	0	PRL	1	1
301	2	38	Female	23.7	1	23.0	1	0	NF	1	1
303	2	44	Female	24.2	0	14.0	1	0	PRL	1	1
340	1	34	Female	27.9	1	18.0	1	0	NF	1	1
345	2	55	Female	31.2	1	30.0	1	1	NF	0	0
349	2	31	Male	22.0	0	27.2	1	1	NF	1	0
363	1	18	Female	27.7	0	15.7	1	0	GH	1	1
369	2	57	Female	28.3	0	14.0	1	0	NF	0	1
398	2	65	Male	23.8	0	19.0	1	0	NF	0	1
405	2	62	Male	31.0	1	35.0	1	1	GH	1	0

© 2022 Guo X et al. *JAMA Network Open.*

eTable 2. List of the Patients With a Secondary Event

Pt no.	Trial Group	Age	Sex	BMI	Medical history, 1=yes, 0=no	Tumor maximal diameter, mm	Macroadenoma, 1=yes, 0=no	Cavernous sinus invasion, 1=yes, 0=no	Tumor type	Hypopituitarism at baseline, 1=yes, 0=no	Gross total resection, 1=yes, 0=no
38	2	58	Male	23.7	1	39.0	1	1	NF	1	0
43	1	47	Female	23.6	1	7.0	0	0	PRL	0	1
46	1	49	Female	23.5	1	12.0	1	0	GH	1	1
97	2	39	Female	23.4	1	25.0	1	1	NF	1	0
127	2	47	Female	23.0	1	13.0	1	0	NF	1	1
166	1	37	Male	29.7	1	28.0	1	1	NF	1	0
173	1	45	Female	29.7	1	36.0	1	0	NF	1	1
199	2	66	Female	26.0	1	21.0	1	0	NF	0	1
217	2	30	Female	25.2	1	11.0	1	0	PRL	0	1
240	1	61	Male	25.9	1	20.0	1	0	NF	1	1
251	2	27	Female	23.7	0	8.0	0	0	PRL	0	1
268	2	38	Female	25.7	1	13.0	1	0	GH	0	1
293	1	69	Male	29.7	1	21.5	1	0	NF	1	1
299	1	36	Female	23.0	0	22.0	1	1	NF	0	0
369	2	57	Female	28.3	0	14.0	1	0	NF	0	1

© 2022 Guo X et al. *JAMA Network Open.*

eTable 3. Subgroup Analyses of the Primary Outcome in the Intention-to-Treat Population

	Hydrocortisone Group	No Hydrocortisone Group	Risk Difference (95% CI)
	patients with events/total, %, (95% CI)		
**AI during perioperation (before POD3)**	**14/218, 6.4%, (3.2% to 9.7%)**	**24/218, 11.0%, (6.9% to 15.2%)**	**+4.6% (-0.7% to 9.9%)**
Age			
Age < 45 years (n=214)	8/105, 7.6%, (2.5% to 12.7%)	12/109, 11.0%, (5.1% to 16.9%)	+3.4% (-4.4% to 11.2%)
Age ≥ 45 years (n=222)	6/113, 5.3%, (1.2% to 9.4%)	12/109, 11.0%, (5.1% to 16.9%)	+5.7% (-1.5% to 12.9%)
Sex			
Male sex (n=172)	6/82, 7.3%, (1.7% to 13.0%)	12/90, 13.3%, (6.3% to 20.4%)	+6.0% (-3.0% to 15.0%)
Female sex (n=264)	8/136, 5.9%, (1.9% to 9.8%)	12/128, 9.4%, (4.3% to 14.4%)	+3.5% (-2.9% to 9.9%)**†**
Body mass index			
Body mass index < 25.5 kg/m^2 (n=235)	3/121, 2.5%, (0% to 5.2%)	14/114, 12.3%, (6.3% to 18.3%)	+9.8% (3.2% to 16.4%)**§**
Body mass index ≥ 25.5 kg/m^2 (n=201)	11/97, 11.3%, (5.0% to 17.7%)	10/104, 9.6%, (3.9% to 15.3%)	-1.7% (-10.2% to 6.8%)**†**
Preoperative comorbidities			
Yes (n=318)	9/161, 5.6%, (2.0% to 9.1%)	13/157, 8.3%, (4.0% to 12.6%)	+2.7% (-2.9% to 8.3%)**†**
No (n=118)	5/57, 8.8%, (1.4% to 16.1%)	11/61, 18.0%, (8.4% to 27.7%)	+9.3% (-2.9% to 21.4%)
Tumor size			
Macroadenoma (n=396)	13/200, 6.5%, (3.1% to 9.9%)	22/196, 11.2%, (6.8% to 15.6%)	+4.7% (-0.9% to 10.3%)
Microadenoma (n=40)	1/18, 5.6%, (0% to 16.1%)	2/22, 9.1%, (0% to 21.1%)	+3.5% (-12.5% to 19.5%)
Cavenous sinus invasion			
Yes (n=158)	3/76, 3.9%, (0% to 8.3%)	8/82, 9.8%, (3.3% to 16.2%)	+5.8% (-2.0% to 13.6%)
No (n=278)	11/142, 7.7%, (3.3% to 12.1%)	16/136, 11.8%, (6.3% to 17.2%)	+4.0% (-3.0% to 11.0%)
Tumor types			
Non-functioning (n=261)	9/127, 7.1%, (2.6% to 11.5%)	16/134, 11.9%, (6.4% to 17.4%)	+4.9% (-2.2% to 11.9%)
Functioning (n=175)	5/91, 5.5%, (0.8% to 10.2%)	8/84, 9.5%, (3.2% to 15.8%)	+4.0% (-3.8% to 11.9%)
Preoperative hormone deficiency			
Yes (n=234)	7/128, 5.5%, (1.5% to 9.4%)	13/106, 12.3%, (6.0% to 18.5%)	+6.8% (-0.6% to 14.2%)
No (n=202)	7/90, 7.8%, (2.2% to 13.3%)	11/112, 9.8%, (4.3% to 15.3%)	+2.0% (-5.8% to 9.9%)**†**
Degree of tumor resection			
Gross-total (n=282)	11/146, 7.5%, (3.3% to 11.8%)	16/136, 11.8%, (6.3% to 17.2%)	+4.2% (-2.7% to 11.1%)
Subtotal (n=154)	3/72, 4.2%, (0% to 8.8%)	8/82, 9.8%, (3.3% to 16.2%)	+5.6% (-2.3% to 13.5%)

† Indicates that "No Hydrocortisone Group" is non-inferior to "Hydrocortisone Group" if judged by the margin of 0.1.

§ Indicates that "Hydrocortisone Group" is superior to "No Hydrocortisone Group".

© 2022 Guo X et al. *JAMA Network Open.*

AI, adrenal insufficiency; POD3, the 3rd postoperative day; 95%CI, 95% confidence interval.

© 2022 Guo X et al. *JAMA Network Open.*

eTable 4. Subgroup Analyses of the Secondary Outcome in the Intention-to-Treat Population

	Hydrocortisone Group	No Hydrocortisone Group	Risk Difference (95% CI)
	patients with events/total, %, (95% CI)		
**AI from POD3 to POM3**	**7/218, 3.2%, (0.9% to 5.6%)**	**8/218, 3.7%, (1.2% to 6.2%)**	**+0.5% (-3.0% to 3.9%)†**
Age			
Age < 45 years (n=214)	2/105, 1.9%, (0% to 4.5%)	4/109, 3.7%, (0.1% to 7.2%)	+1.8% (-2.6% to 6.2%)†
Age ≥ 45 years (n=222)	5/113, 4.4%, (0.6% to 8.2%)	4/109, 3.7%, (0.1% to 7.2%)	-0.8% (-5.9% to 4.4%)†
Sex			
Male sex (n=172)	3/82, 3.7%, (0% to 7.7%)	1/90, 1.1%, (0% to 3.3%)	-2.5% (-7.2% to 2.1%)†
Female sex (n=264)	4/136, 2.9%, (0.1% to 5.8%)	7/128, 5.5%, (1.5% to 9.4%)	+2.5% (-2.3% to 7.4%)†
Body mass index			
Body mass index < 25.5 kg/m^2 (n=235)	3/121, 2.5%, (0% to 5.2%)	5/114, 4.4%, (0.6% to 8.1%)	+1.9% (-2.8% to 6.6%)†
Body mass index ≥ 25.5 kg/m^2 (n=201)	4/97, 4.1%, (0.2% to 8.1%)	3/104, 2.9%, (0% to 6.1%)	-1.2% (-6.3% to 3.9%)†
Preoperative comorbidities			
Yes (n=318)	6/161, 3.7%, (0.8% to 6.7%)	6/157, 3.8%, (0.8% to 6.8%)	+0.1% (-4.1% to 4.3%)†
No (n=118)	1/57, 1.8%, (0% to 5.2%)	2/61, 3.3%, (0% to 7.7%)	+1.5% (-4.1% to 7.1%)†
Tumor size			
Macroadenoma (n=396)	6/200, 3.0%, (0.6% to 5.4%)	7/196, 3.6%, (1.0% to 6.2%)	+0.6% (-2.9% to 4.1%)†
Microadenoma (n=40)	1/18, 5.6%, (0% to 16.1%)	1/22, 4.5%, (0% to 13.2%)	-1.0% (-14.7% to 12.7%)
Cavernous sinus invasion			
Yes (n=158)	2/76, 2.6%, (0% to 6.2%)	2/82, 2.4%, (0% to 5.8%)	-0.2% (-5.1% to 4.7%)†
No (n=278)	5/142, 3.5%, (0.5% to 6.6%)	6/136, 4.4%, (1.0% to 7.9%)	+0.9% (-3.7% to 5.5%)†
Tumor types			
Non-functioning (n=261)	5/127, 3.9%, (0.6% to 7.3%)	5/134, 3.7%, (0.5% to 6.9%)	-0.2% (-4.9% to 4.5%)†
Functioning (n=175)	2/91, 2.2%, (0% to 5.2%)	3/84, 3.6%, (0% to 7.5%)	+1.4% (-3.6% to 6.4%)†
Preoperative hormone deficiency			
Yes (n=234)	5/128, 3.9%, (0.5% to 7.3%)	3/106, 2.8%, (0% to 6.0%)	-1.1% (-5.7% to 3.5%)†
No (n=202)	2/90, 2.2%, (0% to 5.3%)	5/112, 4.5%, (0.6% to 8.3%)	+2.2% (-2.6% to 7.1%)†
Degree of tumor resection			
Gross-total (n=282)	5/146, 3.4%, (0.5% to 6.4%)	6/136, 4.4%, (1.0% to 7.9%)	+1.0% (-3.6% to 5.5%)†
Subtotal (n=154)	2/72, 2.8%, (0% to 6.6%)	2/82, 2.4%, (0% to 5.8%)	-0.3% (-5.4% to 4.7%)†

† Indicates that "No Hydrocortisone Group" is non-inferior to "Hydrocortisone Group" if judged by the margin of 0.1.

AI, adrenal insufficiency; POD3, the 3rd postoperative day; POM3, the 3rd postoperative month; 95%CI, 95% confidence interval.

© 2022 Guo X et al. *JAMA Network Open.*

**eTable 5.** Prognostic Factors Associated With a Primary Event in 436 Patients			
	**Patients with Event (n=38)**	**Patients without Event (n=398)**	**P Value**
Sex			
Male sex (n=172)	18	154	Reference
Female sex (n=264)	20	244	0.296
Age			
Absolute values - year	43.7 ± 11.3	45.1 ± 13.6	0.474
Age < 45 years (n=214)	20	194	Reference
Age ≥ 45 years (n=222)	18	204	0.647
Body mass index			
Absolute values - kg/m2	26.1 ± 3.7	25.4 ± 3.7	0.273
Body mass index < 25.5 kg/m2 (n=235)	17	218	Reference
Body mass index ≥ 25.5 kg/m2 (n=201)	21	180	0.236
Preoperative comorbidities			
No (n=118)	16	102	Reference
Yes (n=318)	22	296	0.029
Hypertension (n=115)	9	106	0.157
Diabetes mellitus (n=44)	3	41	0.236
Deep vein thrombosis (n=2)	0	2	1.000
Bone mineral density loss (n=63)	5	58	0.260
Tumor size			
Absolute values - mm	21.5 ± 9.0	20.5 ± 8.2	0.462
Microadenoma (n=40)	3	37	Reference
Macroadenoma (n=396)	35	361	1.000
Cavernous sinus invasion			
No (n=278)	27	251	Reference
Yes (n=158)	11	147	0.328
Tumor types			
Non-functioning (n=261)	25	236	Reference
Functioning (n=175)	13	162	0.435
GH-secreting (n=101)	8	93	0.623
Prolactin-secreting (n=62)	5	57	0.712
Preoperative serum hormone levels			
ACTH - pg/ml	31.0 ± 19.5	29.8 ± 19.9	0.720
Cortisol - ug/dl	10.4 ± 5.8	13.6 ± 5.6	**0.001***
<9.25 ug/dl (n=102)	17	85	Reference
≥9.25 ug/dl (n=334)	21	313	**0.001***
Preoperative hormone deficiency			
No (n=202)	18	184	Reference
Yes (n=234)	20	214	0.893

© 2022 Guo X et al. *JAMA Network Open.*

Hypothyroidism (n=139)	11	128	0.764
Hypogonadism (n=135)	13	122	0.823
GH deficiency (n=20)	5	15	0.062
Degree of tumor resection			
Subtotal (n=154)	11	143	Reference
Gross-total (n=282)	27	255	0.390
* Indicates statistically significant differences after being justified by the FDR algorithm, used to control the chance of generating false positives during multiple comparisons.			
ACTH, adrenocorticotropic hormone; GH, growth hormone.			

© 2022 Guo X et al. *JAMA Network Open.*

eTable 6. Prognostic Factors Associated With a Secondary Event in 436 Patients

	Patients with Event (n=15)	Patients without Event (n=421)	P Value
Sex			
Male sex (n=172)	4	168	Reference
Female sex (n=264)	11	253	0.303
Age			
Absolute values - year	47.1 ± 12.9	44.9 ± 13.4	0.538
Age < 45 years (n=214)	6	208	Reference
Age ≥ 45 years (n=222)	9	213	0.474
Body mass index			
Absolute values - kg/m2	25.6 ± 2.6	25.4 ± 3.7	0.859
Body mass index < 25.5 kg/m2 (n=235)	8	227	Reference
Body mass index ≥ 25.5 kg/m2 (n=201)	7	194	0.964
Preoperative comorbidities			
No (n=118)	3	115	Reference
Yes (n=318)	12	306	0.531
Hypertension (n=115)	2	113	1.000
Diabetes mellitus (n=44)	0	44	0.563
Deep vein thrombosis (n=2)	0	2	1.000
Bone mineral density loss (n=63)	3	60	0.720
Tumor size			
Absolute values - mm	19.4 ± 9.6	20.6 ± 8.2	0.565
Microadenoma (n=40)	2	38	Reference
Macroadenoma (n=396)	13	383	0.910
Cavernous sinus invasion			
No (n=278)	11	267	Reference
Yes (n=158)	4	154	0.433
Tumor types			
Non-functioning (n=261)	10	251	Reference
Functioning (n=175)	5	170	0.584
GH-secreting (n=101)	2	99	0.579
Prolactin-secreting (n=62)	3	59	0.997
Preoperative serum hormone levels			
ACTH - pg/ml	24.5 ± 15.5	30.1 ± 20.0	0.285
Cortisol - ug/dl	7.7 ± 5.5	13.5 ± 5.6	**<0.001***
<8.8 ug/dl (n=96)	10	86	Reference
≥8.8 ug/dl (n=340)	5	335	**<0.001***
Preoperative hormone deficiency			
No (n=202)	7	195	Reference
Yes (n=234)	8	226	0.979

© 2022 Guo X et al. *JAMA Network Open.*

Hypothyroidism (n=139)	7	132	0.473
Hypogonadism (n=135)	3	132	0.740
GH deficiency (n=20)	4	16	0.011
Degree of tumor resection			
Subtotal (n=154)	4	150	Reference
Gross-total (n=282)	11	271	0.475
* Indicates statistically significant differences after being justified by the FDR algorithm, used to control the chance of generating false positives during multiple comparisons.			
ACTH, adrenocorticotropic hormone; GH, growth hormone.			

© 2022 Guo X et al. *JAMA Network Open.*

eTable 7. Adjusted Incidence Rate Difference and 95% CI for the Primary Outcome

	No-Hydrocortisone Group	Hydrocortisone Group	Risk Difference (95% CI)
	patients with events/total, %, (95% CI)		
**Unadjusted Primary Outcome**			
New-onset AI during perioperation	24/218, 11.0%, (6.9%, 15.2%)	14/218, 6.4%, (3.2%, 9.7%)	**+4.6% (-0.7%, 9.9%) †**
BMI<25.5 kg/m^2	14/114, 12.3%, (6.3% to 18.3%)	3/121, 2.5%, (0% to 5.2%)	**+9.8% (3.2% to 16.4%) §**
BMI≥25.5 kg/m^2	10/104, 9.6%, (3.9% to 15.3%)	11/97, 11.3%, (5.0% to 17.7%)	**-1.7% (-10.2% to 6.8%) †**
**Adjusted Primary Outcome #**			
New-onset AI during perioperation	-	-	**+4.4% (-0.8%, 9.8%) †**

† Indicates that "No-Hydrocortisone Group" is non-inferior to "Hydrocortisone Group" if judged by the margin of 0.1.
§ Indicates that "No-Hydrocortisone Group" is inferior to "Hydrocortisone Group".
# The analysis was adjusted with Cochran-Mantel-Haenszel weighting for preoperative baseline cortisol level and body mass index.

© 2022 Guo X et al. *JAMA Network Open.*

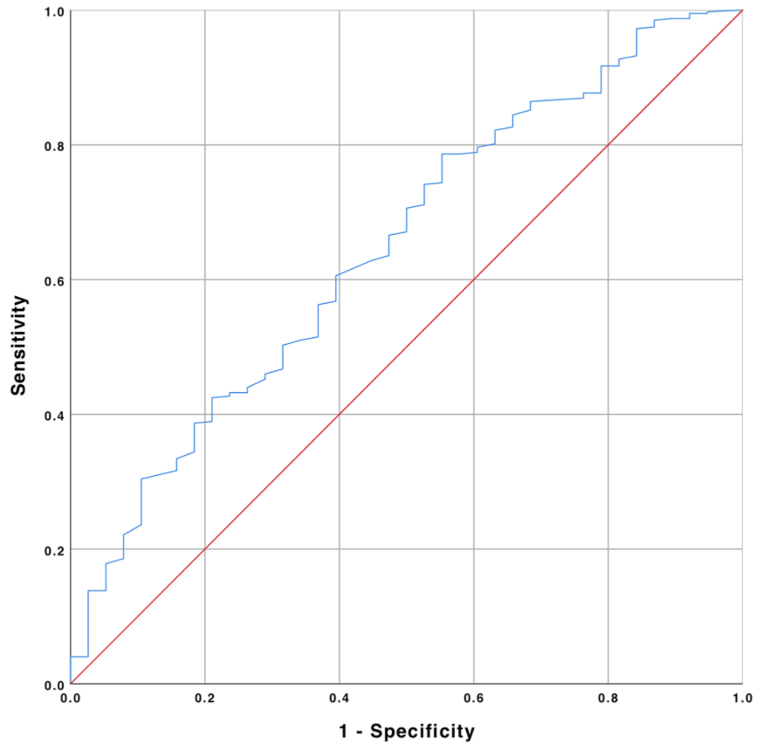

eFigure 1. ROC Curve for Preoperative Cortisol in Predicting AI Before POD3 After Surgery

The cut-off value was estimated as 9.25, with a sensitivity of 0.786 and (1-specificity) of 0.553. AI, adrenal insufficiency; POD3, the 3rd postoperative day; ROC, receiver operator characteristic.

© 2022 Guo X et al. *JAMA Network Open.*

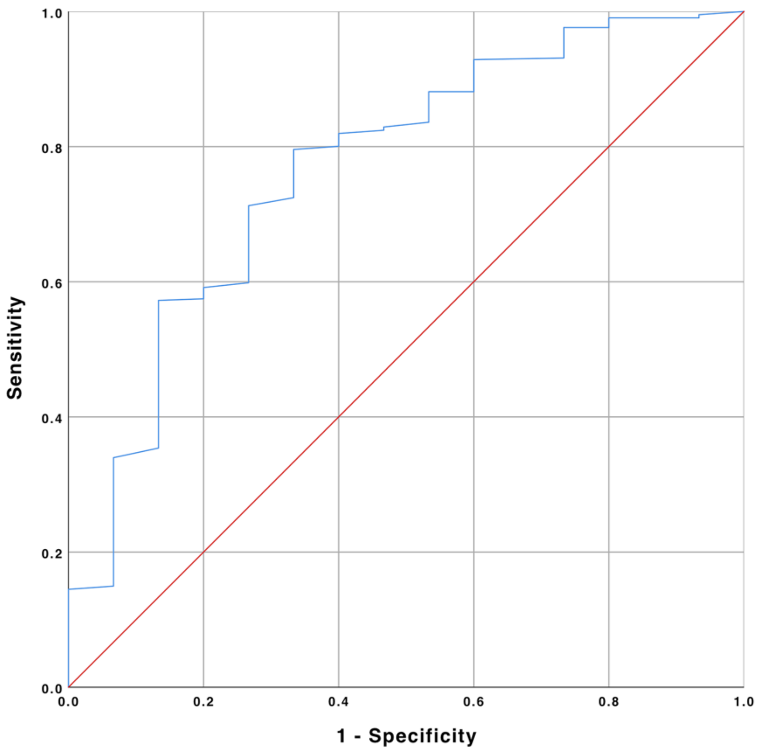

eFigure 2. ROC Curve for Preoperative Cortisol in Predicting AI From POD3 to POM3

The cut-off value was estimated as 8.8, with a sensitivity of 0.796 and (1-specificity) of 0.333. AI, adrenal insufficiency; POD3, the 3rd postoperative day; POM3, the 3rd postoperative month; ROC, receiver operator characteristic.

© 2022 Guo X et al. *JAMA Network Open.*
