# Supplementary material for: Safety of Withholding Perioperative Hydrocortisone for Patients With Pituitary Adenomas With an Intact Hypothalamus-Pituitary-Adrenal Axis: A Randomized Clinical Trial
Source: JAMA Netw Open. 2022 Nov 16;5(11):e2242221. doi: 10.1001/jamanetworkopen.2022.42221 (PMC9669812; doi:10.1001/jamanetworkopen.2022.42221)
Supplement: Supplement 3. — Nonauthor Collaborators [file jamanetwopen-e2242221-s003.pdf]

Supplemental Online Content: Nonauthor Collaborators
*First name, last name, and suffix (if applicable) are required and will appear in PubMed.

*Group Name(s): The ZS-2608 Trial Team							
***First Name and Middle Initial(s)**	***Last Name**	***Suffix (eg, Jr, III)**	Academic Degrees	Institution	Location (city, state/province, country)	Role or Contribution, eg, chair, principal investigator	Group (if more than 1 Group listed in the byline) and/or Subgroup (eg, Steering Committee)
Xiaolin	Xu		MD	Peking Union Medical College Hospital	Beijing, China	allocating drugs/placeboes and maintaining masking	the ZS-2608 Trial Team
Xiang'an	Wu		MD	Peking Union Medical College Hospital	Beijing, China	allocating drugs/placeboes and maintaining masking	the ZS-2608 Trial Team
Yongxing	Xue		MD	Peking Union Medical College Hospital	Beijing, China	allocating drugs/placeboes and maintaining masking	the ZS-2608 Trial Team
Xinyu	Jia		MD	Peking Union Medical College Hospital	Beijing, China	allocating drugs/placeboes and maintaining masking	the ZS-2608 Trial Team
Yajie	Tian		MD	Peking Union Medical College Hospital	Beijing, China	allocating drugs/placeboes and maintaining masking	the ZS-2608 Trial Team
Ke	Zhao		MD	Peking Union Medical College Hospital	Beijing, China	allocating drugs/placeboes and maintaining masking	the ZS-2608 Trial Team
Zhengyun	Liang		MD	Peking Union Medical College Hospital	Beijing, China	allocating drugs/placeboes and maintaining masking	the ZS-2608 Trial Team
Mingzi	Zhang		MD	Peking Union Medical College Hospital	Beijing, China	allocating drugs/placeboes and maintaining masking	the ZS-2608 Trial Team
Zonglin	Huang		MD	Peking Union Medical College Hospital	Beijing, China	allocating drugs/placeboes and maintaining masking	the ZS-2608 Trial Team
Junsheng	Leng		MD	Peking Union Medical College Hospital	Beijing, China	allocating drugs/placeboes and maintaining masking	the ZS-2608 Trial Team
Xuewei	Zhong		MD	Peking Union Medical College Hospital	Beijing, China	allocating drugs/placeboes and maintaining masking	the ZS-2608 Trial Team
Zijun	Zhao		MD	Peking Union Medical College Hospital	Beijing, China	allocating drugs/placeboes and maintaining masking	the ZS-2608 Trial Team
Yi'ou	Wang		MD	Peking Union Medical College Hospital	Beijing, China	allocating drugs/placeboes and maintaining masking	the ZS-2608 Trial Team
Jingcheng	Zhang		MD	Peking Union Medical College Hospital	Beijing, China	allocating drugs/placeboes and maintaining masking	the ZS-2608 Trial Team
Xiaohua	Yang		MD	Peking Union Medical College Hospital	Beijing, China	allocating drugs/placeboes and maintaining masking	the ZS-2608 Trial Team
Miao	Dong		MD	Peking Union Medical College Hospital	Beijing, China	allocating drugs/placeboes and maintaining masking	the ZS-2608 Trial Team
