# Supplementary material for: Safety of Withholding Perioperative Hydrocortisone for Patients With Pituitary Adenomas With an Intact Hypothalamus-Pituitary-Adrenal Axis: A Randomized Clinical Trial
Source: JAMA Netw Open. 2022 Nov 16;5(11):e2242221. doi: 10.1001/jamanetworkopen.2022.42221 (PMC9669812; doi:10.1001/jamanetworkopen.2022.42221)
Supplement: Supplement 4. — Data Sharing Statement [file jamanetwopen-e2242221-s004.pdf]

Data Sharing Statement

Guo. Safety of Withholding Perioperative Hydrocortisone for Patients With Pituitary Adenomas With an Intact Hypothalamus-Pituitary-Adrenal Axis. *JAMA Netw Open*. Published November 16, 2022. doi:10.1001/jamanetworkopen.2022.42221

Data
Data available: Yes
Data types: Deidentified participant data
How to access data: The dataset can be accessed by academic researchers by contacting the corresponding authors: Wei Lian (lwpumch@hotmail.com) and Bing Xing (xingbingemail@aliyun.com).
When available: With publication

Supporting Documents
Document types: Statistical/analytic code, Informed consent form
How to access documents: The statistical/analytic code and informed consent form can be accessed by academic researchers by contacting the corresponding authors: Wei Lian (lwpumch@hotmail.com) and Bing Xing (xingbingemail@aliyun.com).
When available: With publication

Additional Information
Who can access the data: Researchers who need the data for further scientific analyses.
Types of analyses: For research/study purpose.
Mechanisms of data availability: After approval of a proposal.
